# A Green Blue LED-Driven Two-Liquid-Phase One-Pot Procedure for the Synthesis of Estrogen-Related Quinol Prodrugs

**DOI:** 10.3390/molecules27248961

**Published:** 2022-12-16

**Authors:** Elisa De Marchi, Lorenzo Botta, Bruno Mattia Bizzarri, Raffaele Saladino

**Affiliations:** Department of Biological and Ecological Sciences, University of Tuscia, 01100 Viterbo, Italy

**Keywords:** estrogens, estrogen-related quinols, prodrug, CNS-selective estrogen therapy, DHED, HEDD, neuroprotection, para-quinol, photo-oxygenation, photochemistry

## Abstract

Quinol derivatives of estrogens are effective pro-drugs in steroid replacement therapy. Here, we report that these compounds can be synthesized in one-pot conditions and high yield by blue LED-driven photo-oxygenation of parent estrogens. The oxidation was performed in buffer and eco-certified 2-methyltetrahydrofuran as the two-liquid-phase reaction solvent, and in the presence of meso-tetraphenyl porphyrin as the photosensitizer. Two steroidal prodrugs 10β, 17β-dihydroxyestra-1,4-dien-3-one (DHED) and 10β-Hydroxyestra-1,4-diene-3,17-dione (HEDD) were obtained with high yield and selectivity.

## 1. Introduction

**Figure 1:**
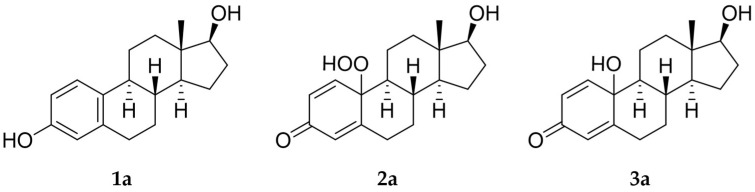
Structures of 17β-estradiol **1a**, hydro-peroxide **2a** and 10β, 17β-dihydroxyestra-1,4-dien-3-one (DHED) **3a**.

Estrogens are steroidal hormones characterized by a variety of biological effects, including anti-cancer activity, prevention of heart diseases, and neuroprotection [1]. In addition, they are applied in Hormone Replacement Therapy (HRT) for the prevention of chronic diseases in post-menopausal women. Unfortunately, current estrogen therapy is limited due to the presence of undesired side effects [2,3,4], such as increased risk of breast cancer [5], thromboembolism, coronary heart disease, and stroke [6,7]. As a result, analogues of estrogens are required to counteract the side effects. Quinol derivatives of estrogens are effective pro-drugs for this HRT. They are converted to corresponding estrogens in the brain, remaining inactive in the rest of the body. This allows the efficient treatment of neurological and psychiatric diseases, without emergence of peripheral side effects [3,8,9]. In this framework, 10β, 17β-dihydroxyestra-1,4-dien-3-one (DHED), received a particular attention as an alternative to 17β-estradiol. In vitro and in vivo studies showed that DHED has the potential to treat menopausal symptoms [9], ocular neurodegenerations (including glaucoma) [10,11], androgen deprivation-associated hot flushes [12], and Alzheimer’s [13] and Parkinson’s neurological disorders [14]. Estrogen-related quinols are synthesized by the oxygenation of the phenolic A-ring of the molecule [15,16,17,18,19,20,21], the procedure being limited by the use of stoichiometric oxidants (e.g., oxone [22], hypervalent iodine [23,24], and excess of hydrogen peroxide H_2_O_2_ [25]). As an alternative, dye-sensitized photo-oxygenation of 17β-estradiol **1a** has been reported to yield mixtures of the corresponding hydro-peroxide **2a** and DHED **3a** [26] (the structures of compounds **1a**, **2a** and **3a** are reported in Figure 1), the selectivity of the oxidation being dependent from the reaction solvent, substitution pattern [27,28], flow conditions [29], and photosensitizer properties. The synthesis of DHED by multi-step chrysazine-triggered photo-oxygenation in the presence of 1,8-dihydroxyanthraquinone (1,8-HOAQ) and PPh_3_ has been also reported [30].

Recently, we described that singlet oxygen produced from blue-LED irradiation of meso-tetraphenyl porphyrin (meso-TPP) can be trapped by 2-methyltetrahydrofuran (2-MeTHF), favoring the oxidative coupling of phenols by Horseradish Peroxidase (HRP) [31]. This procedure avoided the inactivation of HRP by excess H_2_O_2_, working under experimental conditions simpler than those for in situ reduction of dioxygen [32,33,34,35,36]. Here, we describe the application of this procedure in the synthesis of estrogen-related hydroperoxide and quinol derivatives. The reaction solvent, photosensitizer, and buffer solution have been optimized in order to obtain high conversion of substrate and yield of the desired product.

## 2. Results and Discussion

**Scheme 1:**
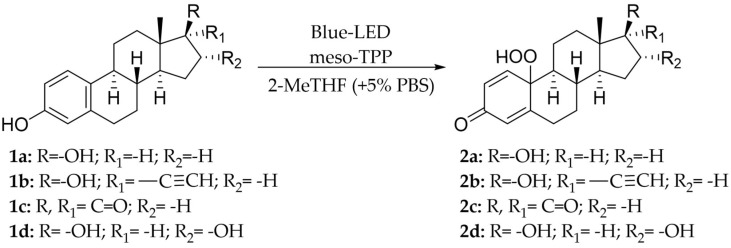
Blue LED−driven two−liquid−phase photo-oxygenation of estrogens **1a**–**d** to hydro-peroxides **2a**–**d**.

17α-Ethinylestradiol **1b** was first studied as a model substrate. Compound **1b** (0.2 mmoL) was dissolved in 2-MeTHF (32 mL) in the presence of meso-TPP (1.0 mol% with respect to substrate), followed by the addition of HRP (407 U) in sodium phosphate buffer (PBS; 16 mL 0.1 M, pH 6.0). The solution was gently stirred (200 rpm) under blue-LED irradiation (blue-LED stripes, 470 nm) and air atmosphere for 24 h at 28 °C. The photoreactor consisted of an internal jar (4.5 cm diameter) inserted in a supplementary external jar (7.5 cm diameter), and blue-LED strips were wrapped around the external jar and covered by aluminum foil (Figure S1). Under these experimental conditions, the hydro-peroxide **2b** was isolated as the only recovered product in low yield, besides the unreacted substrate (Scheme 1; Table 1, entry 1). No trace amounts of dimeric products, possibly derived from oxidative radical homo-coupling processes, were detected in the reaction mixture. The structure of hydro-peroxide **2b** was confirmed by spectroscopic and spectrometric analyses (including 2D NMR analysis; SI-Section 8), and by comparison with data previously reported [37].

When the reaction was carried out in the absence of HRP, the hydro-peroxide **2b** was again obtained as the only recovered product in acceptable yield, suggesting that the enzyme was not involved in the oxidation of the substrate (Table 1, entry 2). In addition, compound **1b** was unreactive when the reaction was carried out in the absence of buffer (Table 1, entry 3), under dark conditions (Table 1, entry 4), and without meso-TPP (Table 1, entry 5) highlighting the key role played by blue-photons, pH, and photosensitizer in the transformation.

The activity of meso-TPP was compared with that of other useful photosensitizers, such as tris(2-phenyl-pyridine) iridium [Ir(ppy)_3_] and Rose Bengal (structure and UV-vis adsorption spectra of the photosensitizers are in Figures S7–S9). As reported in Table 1, meso-TPP showed the highest activity in the photo-oxygenation of compound **1b** (entry 2 versus entries 6 and 7).

The possible formation of 2-MeTHF hydro-peroxide from 2-MeTHF during blue LED irradiation, previously observed by us [31], was evaluated by the pyrogallol assay at different reaction times (1, 2, 4, 6, and 24 h) and in the presence—or alternatively in the absence—of compound **1b**. As reported in Figure S2, compound **1b** lowered the concentration of 2-MeTHF hydro-peroxide, suggesting higher reactivity of compound **1b** with singlet oxygen with respect to the organic solvent. To optimize the photo-oxygenation procedure we analyzed the effect played by the concentration of substrate, the amount of the buffer (and relative pH), and the nature of the reaction solvent, on the process. Correspondingly to the other experimental parameters, hydro-peroxide **2b** was obtained in higher yield starting from 60 mM of substrate (Table 2, entry 1). This result was in accordance with the effect played by the concentration of the substrate on the intensity of the blue LED-photons in the bulk of the solution [38]. The high yield of hydro-peroxide **2b** was retained in the presence of a low amount of buffer (160 µL, 5% *v/v* with respect to 2-MeTHF) (Table 2, entry 2) at pH 6, while it decreased at pH 8 (5% NaHCO_3_ ss; Table 2, entry 3), and at pH 2 (AcOH 0.5%; Table 2, entry 4). Finally, we studied the effect of a panel of reaction solvents, characterized by a different stabilization effect for singlet oxygen, including CH_2_Cl_2_, EtOAc, and HFIP [39]. The highest yield of hydro-peroxide **2b** was obtained in CH_2_Cl_2_ (>98%; Table 2, entry 6) confirming the high stabilizing effect previously reported (Table 2, entry 6 versus entries 2, 5 and 7) [40]. The general order of reactivity was as follows: CH_2_Cl_2_ > 2-MeTHF > HFIP > EtOAc.

Next, we studied the photo-oxygenation of 17β-estradiol **1a**, estrone **1c**, and estriol **1d** under optimal experimental conditions (that is: 60 mM of substrate, CH_2_Cl_2_, PBS, and meso-TPP). Unfortunately, estradiol **1a** and estriol **1d** showed very low conversion of substrate due to the limited solubility of CH_2_Cl_2_, while hydro-peroxide **2c** was obtained in quantitative conversion of the substrate and yield of the product (Table 3, entry 1).

The reaction was successively repeated in the second most reactive organic solvent previously observed in the oxidation of compound **1b**, 2-MeTHF, also taking advantage of its sustainability [35,36]. Under these experimental conditions, hydro-peroxides **2a** and **2c**–**d** were obtained, ranging from acceptable to high yields (Table 3, entry 2 and entries 4–5).

The mechanism of dye-mediated photo-oxygenation of phenols has been reviewed and discussed; it includes the transfer of the excited state from the photosensitizer to the substrate (Type I mechanism), or alternatively the inter-crossing system between the photosensitizer and dioxygen, with formation of singlet oxygen (^1^O_2_) (Type II mechanism) [38,41,42,43]. Under our experimental conditions, the Type I mechanism was most probably not operating, as suggested by the loss of reactivity of the substrate in the absence of meso-TPP (Table 1, entry 5), associated with the low absorption coefficient of estrogens in the interaction with blue-LED photons (Figures S3–S6) [44,45,46,47]. Additional experiments were performed to investigate the possible involvement of the Type II mechanism [43,48]. Hydroxy and superoxide radicals were not produced during the reaction as evaluated by the “coumarin” assay [48,49,50,51] (Figure S10) and the TEMPO assay (Figure S11) [52], respectively, while the NaN_3_ assay (Figure S12) confirmed the involvement of ^1^O_2_ [53,54,55]. In addition, the reaction was not effective under an argon atmosphere in the presence of degassed solvents (SI-Section 6). Although the possibility of the Type I mechanism cannot be completely ruled out, these data support the formation of ^1^O_2_ as the primary oxidant in the photo-oxygenation of estrogens **1a**–**d**. This result is in accordance with the reported ability of meso-TPP to produce ^1^O_2_ in aerated systems [42,43].

**Scheme 2:**
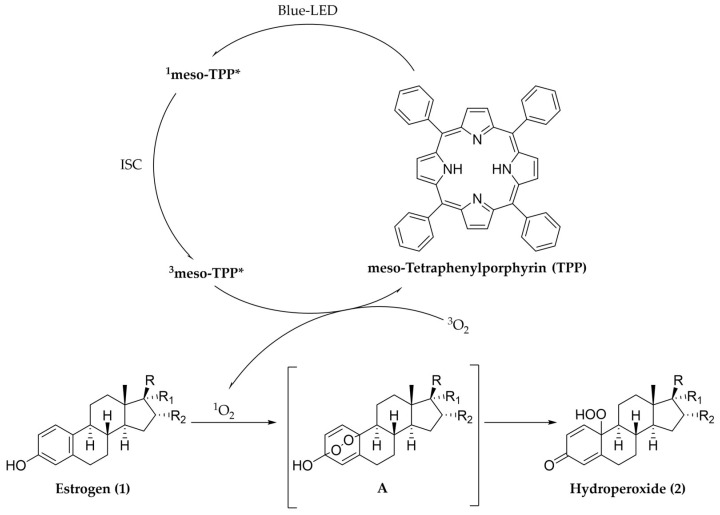
Tentative reaction pathway for the photo-oxygenation of estrogen **1** by blue-LED irradiation in the presence of meso-TPP and bi-phasic system. “*” represents the excited structure of meso-TPP.

The tentative reaction pathway for the photo-oxygenation of compounds **1a**–**d** is reported in Scheme 2, it includes: (i) blue-LED photo-activation of meso-TPP to form the singlet excited state (^1^meso-TPP*); (ii) intersystem crossing (ISC) to form the triplet excited state (^3^meso-TPP*); (iii) energy transfer, and formation of singlet oxygen (^1^O_2_); (iv) selective insertion of ^1^O_2_ on substrate to yield an unstable adduct **A** (not isolated in our case); and (v) rearrangement of adduct **A** to yield the corresponding hydro-peroxide.

**Scheme 3:**
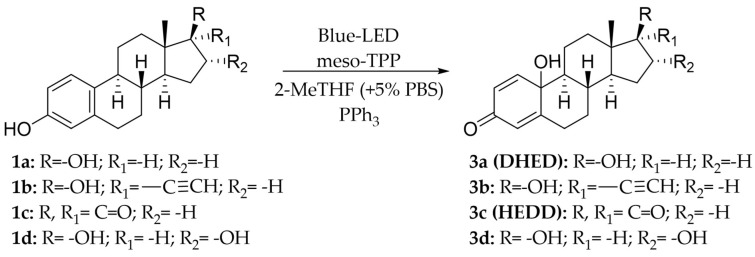
One−pot synthesis of quinols **3a**–**d**.

The reduction of hydro-peroxide **2b** was performed with different redox agents, Na_2_S_2_O_3_, KI, and PPh_3_. In this latter case, the progress of the reduction was monitored by the analysis of the C-10 signal (81.020 ppm) in the ^13^C NMR spectrum of the substrate, due to the high structural similarity between compound **2b**, and the corresponding quinol derivative **3b** (experimental procedures are in SI-Section 7). Among the reagents studied, PPh_3_ afforded quinol **3b** in quantitative yield and conversion of substrate. PPh_3_ was then used for the design of a novel one-pot synthesis of quinol **3b** by contemporary oxidation of 17α-ethinylestradiol **1b**, and in situ reduction of hydro-peroxide **2b** (Scheme 3).

Compound **1b** (60 mM) and meso-TPP (1.0 mol% with respect to substrate) were dissolved in 2-MeTHF, followed by addition of PBS (0.1M, pH 6; 5% with respect to organic solvent). The solution was gently stirred under blue-LED irradiation at 28 °C, and PPh_3_ was added to the reaction mixture at indicated reaction times. The presence of PPh_3_ at the starting point of the reaction totally inhibited the formation of quinol **3b**, with hydro-peroxide **2b** being the only recovered product (Table 4, entry 1), probably due to the fast oxidation of PPh_3_ to triphenyl-phosphinoxide (TPPO). A better result was obtained when PPh_3_ was added to the reaction mixture after 2 h. In this latter case, quinol **3b** was obtained with 50% yield and 70% conversion of substrate (Table 4, entry 2). The addition of PPh_3_ after 3 h further increased the yield of quinol **3b**, and conversion of substrate (Table 4, entry 3). Longer addition times (e.g., 24 h) did not further increase the yield of quinol **3b** (Table 4, entry 4).

The one-pot procedure was then applied to estrogens **1a** and **1c**–**d** assuming 3 h as the optimal reaction time for the addition of PPh_3_. As reported in Table 5, quinols **3a** and **3c**–**d** were obtained ranging from acceptable to high yield (Table 5, entry 1 and entries 3–4). Quinols **3a** (DHED) and **3c** (HEDD) are well recognized pro-drugs in Hormone Replacement Therapy [9,10,11,12,13,14].

## 3. Materials and Methods

### 3.1. General Considerations

Commercially available reagents were used without further purification. Chromatographic separations were performed on Merck silica gel 60 (230–400 mesh). Rf values are referred to TLC carried out on 0.25 mm silica gel plates (F254) using the eluent indicated for column chromatography. All products were dried in high vacuum (10–3 mbar) before characterization. ^1^H NMR, ^13^C NMR, and 2D NMR were recorded on a Bruker Advance DRX400 (400 MHz/100 MHz) spectrometer. Chemical shifts are in parts per million (δ scale) and internally referenced the CD_3_OD signal at δ 3.31 and 49.00 ± 0.01 ppm, respectively. Coupling constants (J) are reported in Hz. UV-visible (UV-vis) spectra were recorded using Cary 60 UV-Vis spectrophotometer, Agilent, Santa Clara, USA. Blue-LED apparatus consisted of a 1.0 m blue-LED strip (wavelength 470 nm, nominal capacity/m 14.4 W) ‘LEDXON MODULAR 9009083 LED.

### 3.2. General Procedure for the Synthesis of Hydro-Peroxides ***2a**–**d***

The selected estrogen (0.2 mmol) and meso-TPP (1 mol%) were dissolved in 2-Me-THF (3.2 mL), followed by the addition of PBS (0.16 mL; 0.1 M, pH 6), and the mixture was gently stirred (200 rpm) under blue-LED irradiation and air atmosphere at 28 ± 1 °C for 24 h. The reaction mixture was washed with brine (3 × 2 mL), dried over sodium sulphate, and evaporated under vacuum. The crude mixture was purified by column chromatography.

10-hydroperoxy-17-hydroxy-13-methyl-6, 7, 8, 9, 10, 11, 12, 13, 14, 15, 16, 17-dodecahydro-3H-cyclopenta[a]phenanthren-3-one (**2a**):

*R_f_* = 0.18 (PE:AcOEt 1:1); oil (yield 60%). ^1^H NMR (400 MHz, CD_3_OD): δ = 7.31 (d, *J* = 10.4 Hz, 1H), 6.24 (dd, *J* = 8, 2.1 Hz, 1H), 6.10 (s, 1H), 3.56 (t, *J* = 8.4 Hz, 1H), 2.80–2.71 (m, 1H), 2.41 (ddd, *J* = 12.4, 4.2, 2.3 Hz, 1H), 2.03–1.81 (m, 5H), 1.76–1.66 (m, 1H), 1.66–1.56 (m, 1H), 1.53–1.44 (m, 1H), 1.34 (qd, *J* = 12.2, 5.8 Hz, 1H), 1.14–0.92 (m, 4H), 0.80 (s, 3H). 13C NMR (100 MHz, CD3OD): δ 186.6 (C3), 166.9 (C5), 151.9 (C1), 129.4 (C2), 123.9 (C4), 81.1 (C17), 80.6 (C10), 55.6 (C9), 49.9 (C14), 42.8 (C13), 36.2 (C12), 35.4 (C8), 33.3 (C7), 31.7 (C6), 29.1 (C16), 23.0 (C15), 22.7 (C11), 10.0 (C18) ppm; ESIMS m/z 327.1 [M + Na]^+^. The spectral data were in accordance with the results previously reported [26].

17-ethynyl-10-hydroperoxy-17-hydroxy-13-methyl-6,7,8,9,10,11,12,13,14,15,16,17-dodecahydro-3H-cyclopenta[a]phenanthren-3-one (**2b**):

*R_f_* = 0.23 (PE:AcOEt 3:2); oil (yield 85%). ^1^H NMR (400 MHz, CD_3_OD): δ = 7.317 (d, *J* = 10 Hz, 1H), 6.29 (dd, *J* = 8, 2 Hz, 1H), 6.11 (s, 1H), 2.86 (s, 1H), 2.81–280 (m, 1H), 2.43–2.40 (m, 1H), 2.21–2.20 (m, 1H), 2.04–1.86 (m, 5H), 1.73–1.66 (m, 3H), 1.49–1.37 (m, 2H), 1.23–1.22 (m, 1H), 1.19–1.06 (m, 1H), 0.89 (s, 3H) ppm. ^13^C NMR (100 MHz, CD_3_OD): δ = 186.6 (C3), 166.7 (C5), 151.8 (C1), 129.5 (C2), 124.0 (C4), 87.0 (C20), 81.0 (C10), 78.6 (C17), 73.4 (C21), 55.2 (C9), 49.4 (C14), 46.6 (C15), 38.2 (C8), 36.0 (C16), 33.4 (C6), 32.3 (C12), 31.6 (C7), 22.7 (C11), 11.6 (C18) ppm. ESIMS m/z 351.1 [M + Na]^+^. The spectral data were in accordance with the results previously reported [26].

10-hydroperoxy-13-methyl-7,8,9,10,11,12,13,14,15,16-decahydro-3H-cyclopenta[a]phenanthrene-3,17(6H)-dione (**2c**):

*R_f_* = 0.21 (PE:AcOEt 3:2); oil (yield 75%). ^1^H NMR (400 MHz, CD_3_OD): δ = 7.30 (d, *J* = 10, 1H), 6.30 (dd, *J* = 8.4, 2, 1H), 6.13 (s, 1H), 2.85–2.84 (m, 1H), 2.49–2.42 (m, 2H), 2.17–1.90 (m, 6H), 1.81–1.76 (m, 1H), 1.68–1.62 (m, 1H), 1.38–1.18 (m, 4H), 0.94 (s, 3H) ppm. ^13^C NMR (100 MHz, CD_3_OD): δ = 221.4 (C17), 186.5 (C3), 166.3 (C5), 151.5 (C1), 129.6 (C2), 124.1 (C4), 80.9 (C10), 55.0 (C9), 50.0 (C14), 35.0 (C15), 34.9 (C8), 32.4 (C16), 31.4 (C6), 30.9 (C12), 22.1 (C7), 21.4 (C11), 12.6 (C18) ppm. ESIMS m/z 325.1 [M + Na]^+^. The spectral data were in accordance with the results previously reported [37].

10-hydroperoxy-16,17-dihydroxy-13-methyl-6,7,8,9,10,11,12,13,14,15,16,17-dodecahydro-3H-cyclopenta[a]phenanthren-3-one (**2d**):

*R_f_* = 0.26 (CH_2_Cl_2_:MeOH 30:3); oil (45%). ^1^H NMR (400 MHz, CD_3_OD): δ = ^1^H NMR (400 MHz, CD_3_OD): δ = 7.22 (d, *J* = 10.4, 1H), 6.14 (dd, *J* = 12,8, 2, 1H), 5.98 (s, 1H), 4.03 (t, *J* = 6, 7.2, 1H), 2.79–2.78 (m, 1H), 2.35 (d, *J* = 11.2, 1H), 2.02–1.90 (m, 3H), 1.85–1.83 (m, 3H), 1.79–1.69 (m, 1H), 1.54–1.48 (m, 1H), 1.35–1.30 (m, 1H), 1.18–1.04 (m, 3H), 0.85 (s, 3H) ppm. ^13^C NMR (100 MHz, CD_3_OD): δ = 186.8 (C3), 168.9 (C5), 153.5 (C1), 126.3 (C2), 121.4 (C4), 88.9 (C17), 78.7 (C10), 77.1 (C16), 55.3 (C9), 43.5 (C13), 36.1 (C12), 34.4 (C8), 33.9 (C7), 33.2 (C6), 31.7 (C15), 21.9 (C11), 11.2 (C18) ppm. ESIMS m/z 343.1 [M + Na]^+^.

### 3.3. General Procedure for the Synthesis of Estrogen-Related Quinols ***3a**–**d***

The selected estrogen (0.2 mmol) and meso-TPP (1.0 mol%) were dissolved in 2-Me-THF (3.2 mL), followed by the addition of PBS (0.16 mL; 0.1 M, pH 6), and the mixture was gently stirred (200 rpm) under blue-LED irradiation and air atmosphere at 28 ± 1 °C for 3 hrs. Then PPh_3_ (0.3 mmol) was added, and the reaction was left under magnetic stirring for 21 h. After washing with brine (3 × 2 mL), the reaction mixture was dried over sodium sulphate, and evaporated under vacuum. The crude mixture was purified by column chromatography.

10,17-dihydroxy-13-methyl-6,7,8,9,10,11,12,13,14,15,16,17-dodecahydro-3H-cyclopenta[a]phenanthren-3-one (**3a, DHED**):

*R_f_* = 0.17 (CH_2_Cl_2_:AcOEt 9:2.5); oil (yield 62%). ^1^H NMR (400 MHz, CD_3_OD): δ = 7.23 (d, *J* = 10.4 Hz, 1H), 6.14 (dd, *J* = 8, 2.1 Hz, 1H), 5.97 (s, 1H), 3.58 (t, *J* = 8.4 Hz, 1H), 2.79–2.78 (m, 1H), 2.34 (ddd, *J* = 12.4, 4.2, 2.3 Hz, 1H), 2.04–1.73 (m, 5H), 1.76–1.66 (m, 1H), 1.706–1.56 (m, 1H), 1.53–1.44 (m, 1H), 1.34 (qd, *J* = 12.2, 5.8 Hz, 1H), 1.14–0.92 (m, 4H), 0.84 (s, 3H). 13C NMR (100 MHz, CD3OD): δ 186.8 (C3), 169.1 (C5), 153.7 (C1), 126.3 (C2), 121.3 (C4), 80.8 (C17), 69.72 (C10), 55.46 (C9), 49.7 (C14), 42.9 (C13), 36.2 (C12), 34.9 (C8), 33.3 (C7), 31.6 (C6), 29.1 (C16), 23.1 (C15), 22.3 (C11), 10.1 (C18) ppm. ESIMS m/z 311.1 [M + Na]^+^. The spectral data were in accordance with the results previously reported [30].

17-ethynyl-10,17-dihydroxy-13-methyl-6,7,8,9,10,11,12,13,14,15,16,17-dodecahydro-3H-cyclopenta[a]phenanthren-3-one (**3b**):

*R_f_* = 0.18 (CH_2_Cl_2_:AcOEt 9:1); oil (yield 84%). ^1^H NMR (400 MHz, CD_3_OD): δ = 7.31 (d, *J* = 10 Hz, 1H), 6.29 (dd, *J* = 8, 2 Hz, 1H), 6.11 (s, 1H), 2.86 (s, 1H), 2.81–2.80 (m, 1H), 2.43–2.40 (m, 1H), 2.21–2.20 (m, 1H), 2.04–1.86 (m, 5H), 1.73–1.66 (m, 3H), 1.49–1.37 (m, 2H), 1.23–1.22 (m, 1H), 1.19–1.06 (m, 1H), 0.89 (s, 3H) ppm. ^13^C NMR (100 MHz, CD_3_OD): δ = 186.8 (C3), 168.9 (C5), 153.5 (C1), 126.3 (C2), 121.4 (C4), 87.2 (C20), 78.7 (C17), 73.4 (C21), 69.6 (C10), 55.1 (C9), 49.2 (C14), 46.7 (C13), 38.3 (C15), 35.5 (C8), 33.3 (C16), 32.2 (C6), 31.7 (C12), 22.8 (C7), 22.3 (C11), 11.7 (C18) ppm. ESIMS m/z 335.1 [M + Na]^+^. The spectral data were in accordance with the results previously reported [56].

10-hydroxy-13-methyl-7,8,9,10,11,12,13,14,15,16-decahydro-3H-cyclopenta[a]phenanthrene-3,17(6H)-dione (**3c, HEDD**):

*R_f_* = 0.36 (PE:AcOEt 1:1); oil (yield 73%). ^1^H NMR (400 MHz, CD_3_OD): δ = 7.23 (d, *J* = 10, 1H), 6.15 (dd, *J* = 8.4, 2, 1H), 6.00 (s, 1H), 2.84–2.83 (m, 1H), 2.48–2.39 (m, 2H), 2.19–1.97 (m, 6H), 1.83–1.79 (m, 1H), 1.69–1.63 (m, 1H), 1.39–1.12 (m, 4H), 0.98 (s, 3H) ppm. ^13^C NMR (100 MHz, CD_3_OD): δ = 221.8 (C17), 186.7 (C3), 168.5 (C5), 153.2 (C1), 126.5 (C2), 121.5 (C4), 69.5 (C10), 55.0 (C9), 49.8 (C14), 35.1 (C15), 34.4 (C8), 32.3 (C16), 31.5 (C6), 30.9 (C12), 21.8 (C7), 21.5 (C11), 12.7 (C18) ppm. ESIMS m/z 309.1 [M + Na]^+^. The spectral data were in accordance with the results previously reported [57].

10,16,17-trihydroxy-13-methyl-6,7,8,9,10,11,12,13,14,15,16,17-dodecahydro-3H-cyclopenta[a]phenanthren-3-one (**3d**):

*R_f_* = 0.26 (CH_2_Cl_2_:MeOH 30:3); oil (yield 46%). ^1^H NMR (400 MHz, CD_3_OD): δ = ^1^H NMR (400 MHz, CD_3_OD): δ = 7.22 (d, *J* = 10.4, 1H), 6.14 (dd, *J* = 12,8, 2, 1H), 5.98 (s, 1H), 4.03 (t, *J* = 6, 7.2, 1H), 2.79–2.78 (m, 1H), 2.35 (d, *J* = 11.2, 1H), 2.02–1.90 (m, 3H), 1.85–1.83 (m, 3H), 1.79–1.69 (m, 1H), 1.54–1.48 (m, 1H), 1.36–1.30 (m, 1H), 1.18–1.04 (m, 3H), 0.86 (s, 3H) ppm. ^13^C NMR (100 MHz, CD_3_OD): δ = 186.8 (C3), 168.9 (C5), 153.5 (C1), 126.3 (C2), 121.4 (C4), 88.9 (C17), 77.1 (C16), 69.6 (C10), 55.3 (C9), 48.4 (C14), 43.5 (C13), 36.1 (C12), 34.4 (C8), 33.9 (C7), 33.2 (C6), 31.7 (C15), 21.9 (C11), 11.2 (C18) ppm. ESIMS m/z 327.1 [M + Na]^+^.

## 4. Conclusions

In conclusion, we developed a novel one-pot approach for the synthesis of estrogen-related quinols by using blue LED-driven photo-oxygenation in a two-liquid-phase system. The reaction proceeded under mild and sustainable conditions, including with a catalytic amount of meso-TPP, eco-certified 2-MeTHF, and buffer as solvents, and PPh_3_ as the reducing agent. The reaction pathway involved blue-LED photo-activation of meso-TPP, and the generation of singlet oxygen (^1^O_2_) (Type II mechanism), followed by oxidation of estrogen to the corresponding hydro-peroxide, and in situ reduction of hydro-peroxide to the desired quinol. Under these experimental conditions, quinols were synthesized ranging from acceptable to very high yield, including two well recognized pro-drugs in Hormone Replacement Therapy, DHED and HEDD. The irrelevance of the Type I mechanism was suggested by the un-reactivity of the system, in the absence of the photosensitizer associated with the low adsorption capacity of estrogens towards blue-LED photons. The presence of 2-MeTHF hydro-peroxide, OH, and superoxide radicals in the reaction pathway was investigated, and ruled-out by means of different specific assays. The additional scopes and applications of this photocatalytic process will be further investigated in our laboratory.

## Figures and Tables

**Table 1:** Blue LED-driven two-liquid-phase photo-oxygenation of 17α-ethinylestradiol **1b** to hydro-peroxide **2b**.

Entry	Light	Solvents	Photosensitizer (1.0 mol%)	Conversion ^a^ (%)	Yield ^a^ (%)
**1**	Blue-LED	2-MeTHF:PBS (2:1)	meso-TPP	20	12
**2 ^b^**	Blue-LED	2-MeTHF:PBS (2:1)	meso-TPP	20	13
**3**	Blue-LED	2-MeTHF	meso-TPP	-	-
**4**	Dark	2-MeTHF:PBS (2:1)	meso-TPP	-	-
**5**	Blue-LED	2-MeTHF:PBS (2:1)	-	-	-
**6**	Blue-LED	2-MeTHF:PBS (2:1)	Ir(ppy)_3_	18	10
**7**	Blue-LED	2-MeTHF:PBS (2:1)	Rose Bengal	19	2

^a^ Conversion of substrate and yield of product were calculated on the basis of starting mmol of substrate; ^b^ Reaction performed under similar experimental conditions in the absence of HRP.

**Table 2:** Optimization experiments for the synthesis of hydro-peroxide **2b** ^a^.

Entry	Solvents	Conversion ^b^	Yield ^b^(%)
**1 ^c^**	2-MeTHF-PBS (2:1)	90	87
**2 ^c^**	2-MeTHF (+5% PBS)	90	85
**3 ^d^**	2-MeTHF (+5% NaHCO_3_ ss)	48	40
**4 ^e^**	2-MeTHF (+5% AcOH aq.0.5%)	38	32
**5 ^c^**	EtOAc (+5% PBS)	65	56
**6 ^c^**	CH_2_Cl_2_ (+5% PBS)	>98	>98
**7 ^c^**	HFIP (+5% PBS)	86	74

^a^ The reaction was performed solubilizing **1b** (0.2 mmol) and meso-TPP (1.0 mol%) in the organic solvent (3.2 mL) followed by the addition of the PBS. The solution was gently stirred (200 rpm) under blue-LED irradiation (blue-LED stripes, 470 nm) at 28 ± 1 °C for 24 h. ^b^ Conversion of substrate and yield of product were calculated on the basis of starting mmol of substrate; ^c^ PBS (0.1 M, pH 6); ^d^ NaHCO_3_ saturated aqueous solution (pH 8); ^e^ AcOH 0.5% aqueous solution (pH 2).

**Table 3:** Substrate scope of novel blue LED-driven two-liquid- phase photo-oxygenation ^a^.

Entry	Cpd	Structure	Conversion ^b^ (%)	Product ^b^ (%)
**1 ^c^**	**1c**	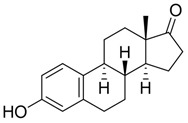	>98%	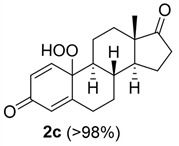
**2**	**1a**	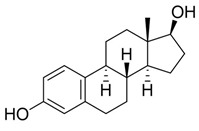	65	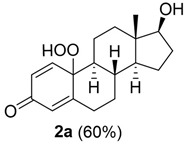
**3**	**1b**	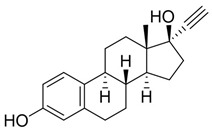	90	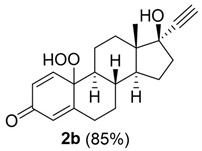
**4**	**1c**	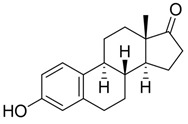	80	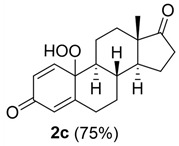
**5**	**1d**	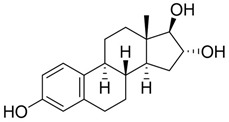	50	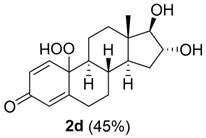

^a^ The reaction was performed solubilizing compound **1** (0.2 mmol) and meso-TPP (1.0 mol%) in 2-MeTHF (3.2 mL), followed by the addition of PBS (160 µL; 0.1M, pH 6). The solution was gently stirred (200 rpm) under blue-LED irradiation (blue-LED stripes, 470 nm) at 28 ± 1 °C for 24 h; ^b^ Conversion of substrate and yield of reaction product were calculated on the basis of starting mmol of substrate; ^c^ Reaction performed in CH_2_Cl_2_.

**Table 4:** One-pot synthesis of quinol **3b** at different times of addition of PPh_3_ ^a^.

Entry	Addition Time (h)	Reaction Time (h)	Conversion ^b^ (%)	Yield ^b^ (%)
**1**	0	24	15	10 ^c^
**2**	2	24	70	50
**3**	3	24	90	84
**4**	24	48	90	88

^a^ The reaction was performed solubilizing **1b** (0.2 mmol) and meso-TPP (1.0 mol%) in 2-MeTHF (3.2 mL), followed by the addition of PBS (0.1 M, pH 6) and of PPh_3_ (0.3 mmol) at different reaction times. The solution was gently stirred (200 rpm) under blue-LED irradiation (blue-LED stripes, 470 nm) at 28 ± 1 °C; ^b^ Conversion of substrate and yield of quinol **3b** were calculated on the basis of mmol of starting substrate. ^c^ Yield of hydro-peroxide **2b**.

**Table 5:** Substrate scope of novel blue LED-driven two-liquid-phase in One-Pot condition ^a^.

Entry	Cpd	Structure	Conversion ^b^ (%)	Product ^b^ (%)
**1**	**1a**	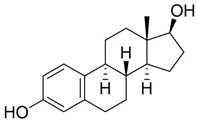	66	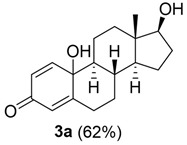
**2**	**1b**	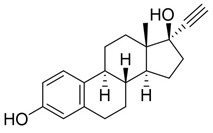	89	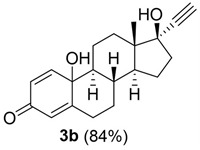
**3**	**1c**	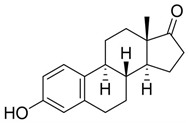	83	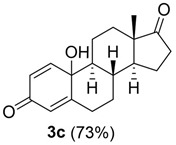
**4**	**1d**	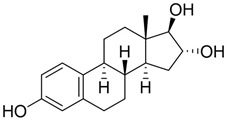	53	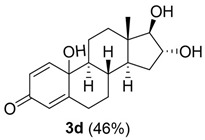

^a^ The reaction was performed solubilizing **1a**–**d** (0.2 mmol) and meso-TPP (1.0 mol%) in 2-MeTHF (3.2 mL) and finally adding PBS (160 µL; 0.1M, pH 6). The solution was gently stirred (200 rpm) under blue-LED irradiation (blue-LED stripes, 470 nm) at 28 ± 1 °C. After 3 h, the PPh_3_ (0.3 mmol) was added to the reaction mixture; ^b^ Conversion of substrate and yield of reaction product were calculated on the basis of mmol of starting substrate.

## Data Availability

Not applicable.

## Supplementary Materials

The following supporting information can be downloaded at: https://www.mdpi.com/article/10.3390/molecules27248961/s1, Reaction set up; 2-Me-THF hydroperoxide assay; UV-visible analysis; Coumarin assay; Quenching experiments; degassed conditions; Screening of reducing agents; 1D and 2D NMR spectra. Figure S1: reaction set up; Figure S2: 2-MeTHF hydroperoxide assay; Figure S3–S6: estrogens UV-visible spectra; Figures S7–S9: photosensitizers UV-visible spectra; Figure S10: coumarin assay; Figure S11: photo-oxygenation of **1c** in presence of TEMPO as radical scavenger; Figure S12: photo-oxygenation of **1c** in presence of NaN_3_ as singlet oxygen scavenger; Figure S13: photo-oxygenation of 1b under degassed conditions; Figure S14: reduction of hydroperoxide **2b** by Na_2_S_2_O_3_; Figure S15: reduction of hydroperoxide **2b** by KI; Figure S16: reduction of hydroperoxide **2b** by PPh_3_; Figures S17–S28: ^1^H NMR, 13C NMR and 2D NMR spectra of hydro-peroxides **2a**–**d**; Figures S29–S36: ^1^H NMR and 13C NMR spectra of quinols **3a**–**d**.
