# Supplementary material for: A Green Blue LED-Driven Two-Liquid-Phase One-Pot Procedure for the Synthesis of Estrogen-Related Quinol Prodrugs
Source: Molecules. 2022 Dec 16;27(24):8961. doi: 10.3390/molecules27248961 (PMC9782465; doi:10.3390/molecules27248961)
Supplement: Supplementary file 1 [file molecules-27-08961-s001.zip › molecules-2069675-supplementary.pdf]

Supporting information

A green blue LED-driven two-liquid- phase One-Pot procedure for the synthesis of Estrogen-related quinol prodrugs

Elisa De Marchi [1], Lorenzo Botta [1], Bruno Mattia Bizzarri, [1] and Raffaele Saladino [1,*]

[1] Department of Biological and Ecological Sciences, University of Tuscia, 01100 Viterbo, Italy; elisa.demarchi@unitus.it (E.D.M.); lorenzo.botta@unitus.it (L.B.); bm.bizzarri@unitus.it (B.M.B.)
* Correspondence: saladino@unitus.it

Table of contents:

1. Reaction setup. S1
2. 2-MeTHF hydroperoxide assay. S2
3. UV-visible analysis of estrogens **1a-d** and photosensitizers. S3
4. Coumarin assay. S6
5. Quenching experiments. S7
   5.1. TEMPO assay S7
   5.2. NaN_3 assay S7
6. Photo-oxygenation of estrogens under argon atmosphere and degassed solvents. S8
7. Screening of reducing agents. S8
   7.1. $Na_2S_2O_3$. S8
   7.2. KI. S9
   7.3. PPh_3. S9
8. NMR data of hydro-peroxides **2a-d** and quinols **3a-d**. S10

1. Reaction setup.

The photoreactor consisted in a 4.5 cm diameter jar inserted in a supplementary 7.5 cm diameter jar (Figure SI1, panel a) with 2 ground glass joints allowing the air supply and the control of the temperature directly inside the reactor (Figure SI1, panel b). Blue-LED strips were wrapped around the external jar and covered by aluminium foil (Figure SI1, panel b). An inlet and an outlet allowed the circulation of refrigerating liquid between external and internal jars through a recirculation system (Figure SI1, panel c). Refrigerating system allowed the set-up of the temperature at 28 ± 1 °C. The blue-LED apparatus consisted in a 1.0-meter blue-led strip (wavelength 470 nm, nominal capacity/m 14.4W) ‘LEDXON MODULAR 9009083 LED, purchased from Farnell Italia SRL (product code 2214013, producer code 9009083).

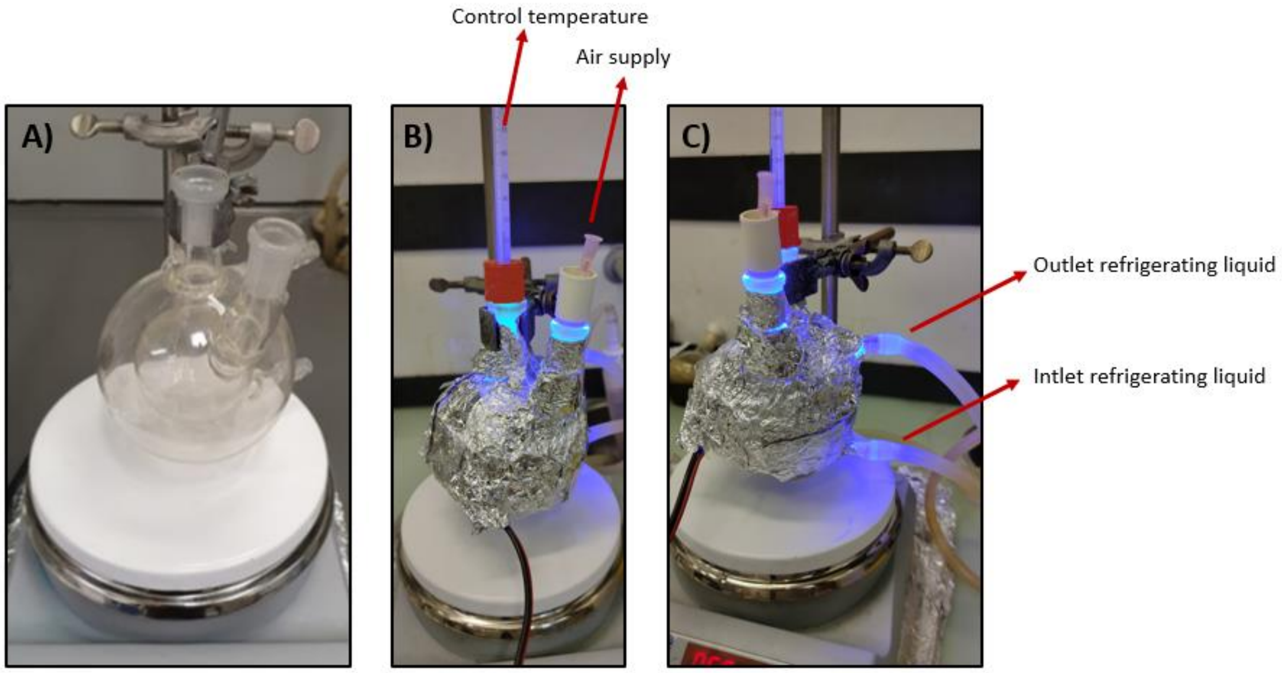

Figure S1: Reaction set up. Panel A): Internal and external jars of the photoreactor. Panel B): The photoreactor wrapped with blue-LED stripes and covered by aluminium foil, control temperature and air supply. Panel C): Inlet and outlet of refrigerating liquid system.

2. 2-MeTHF hydroperoxide assay.

The 2-MeTHF hydroperoxide concentration was evaluated at different times (1, 2, 4, 6 and 24 h) spectrophotometrically by the HRP-pyrogallol assay in the presence (method A) or in the absence (method B) of substrate, using a calibration line made with standard H_2O_2. Method A: 17α-ethinylestradiol **1b** (30 mg, 0.1 mmol) and meso-TPP (0.6 mg, 1.0 mol%) were dissolved in 2-Me-THF (1.6 mL) followed by the addition of PBS (1.6 mL; 0.1M, pH 6) and the mixture was gently stirred (200 rpm) under blue-LED irradiation and air atmosphere. At indicated times, 100 µL of the buffered solution were collected and added to the assay mixture, containing 2.1 mL of water, 380 µL of PBS (0.1 M, pH 6), 320 µL of pyrogallol solution in water (0.4M), and 100 µL of HRP (0.001 U/mL) solution in PBS (0.1 M, pH 6). The oxidation of pyrogallol was followed by the absorbance increase at 420 nm. Method B: meso-TPP (0.6 mg) were dissolved in 2-Me-THF (1.6 mL) followed by the addition of PBS (1.6 mL; 0.1M, pH 6) and the mixture was gently stirred (200 rpm) under blue-LED irradiation and air atmosphere. At indicated times, 100 µL samples of the buffered solution were collected and added to the assay mixture, containing 2.1 mL of water, 380 µL of PBS (0.1 M, pH 6), 320 µL of pyrogallol solution in water (0.4M), and 100 µL of HRP (0.001 U/mL) solution in PBS (0.1 M, pH 6). The oxidation of pyrogallol was followed by the absorbance increase at 420 nm.

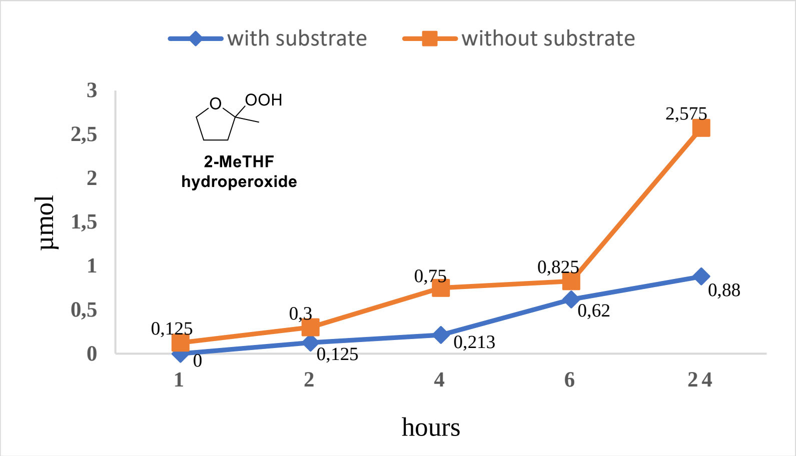

Figure S2: 2-MeTHF hydro-peroxide concentration at different times in presence and absence of substrate.

3. UV-visible analysis of estrogens 1a-d and photosensitizers.

UV-visible analyses were performed at wavelengths from 190 to 800 nm under magnetic stirring at 25°C. The samples were analysed in 2-MeTHF at a concentration of 60 mg/mL. The photosensitiser samples were analysed in 2-MeTHF at a concentration of 20 mg/mL.

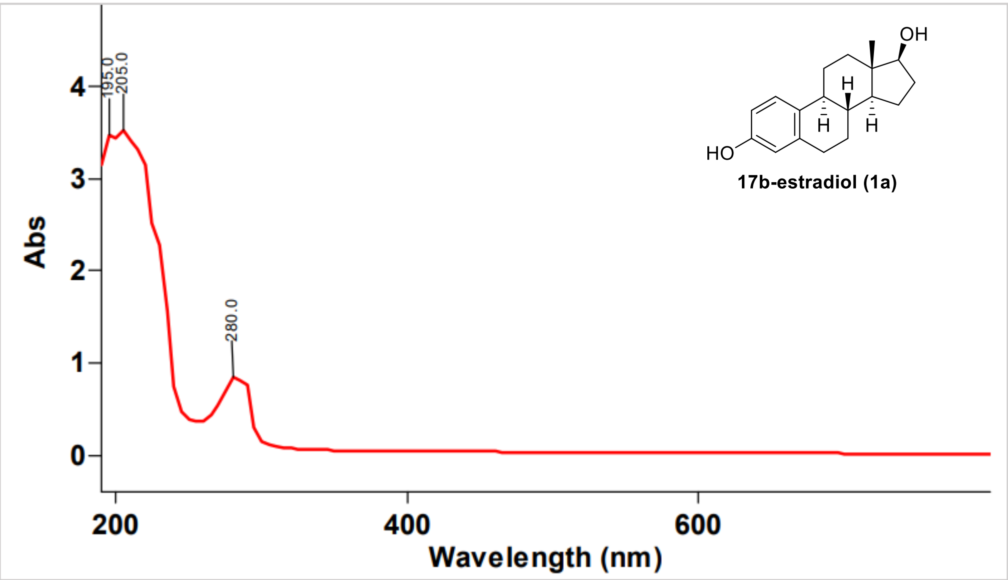

Figure S3: UV-visible spectra of 17β-estradiol **1a** in 2-MeTHF.

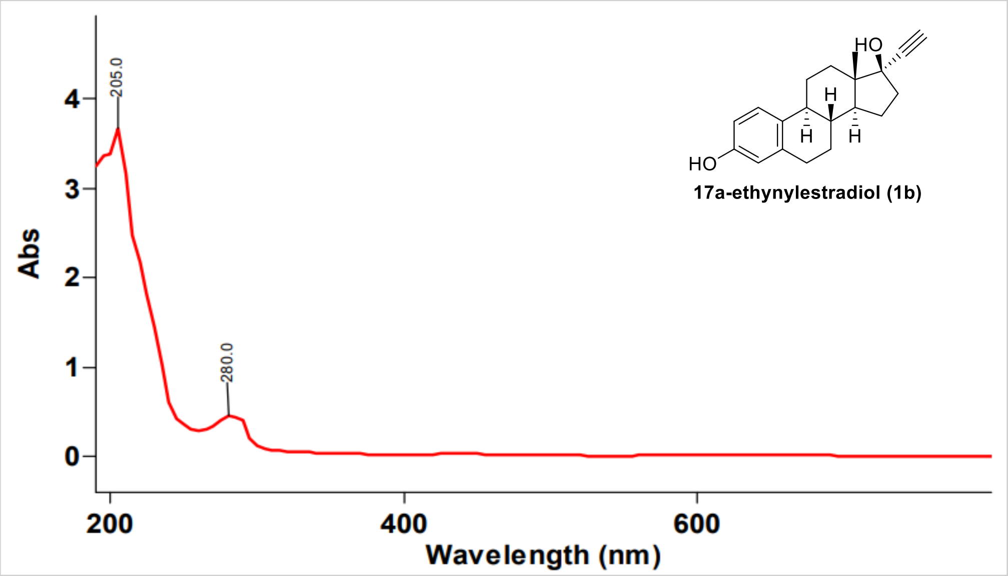

Figure S4: UV-visible spectra of 17α-ethinylestradiol **1b** in 2-MeTHF.

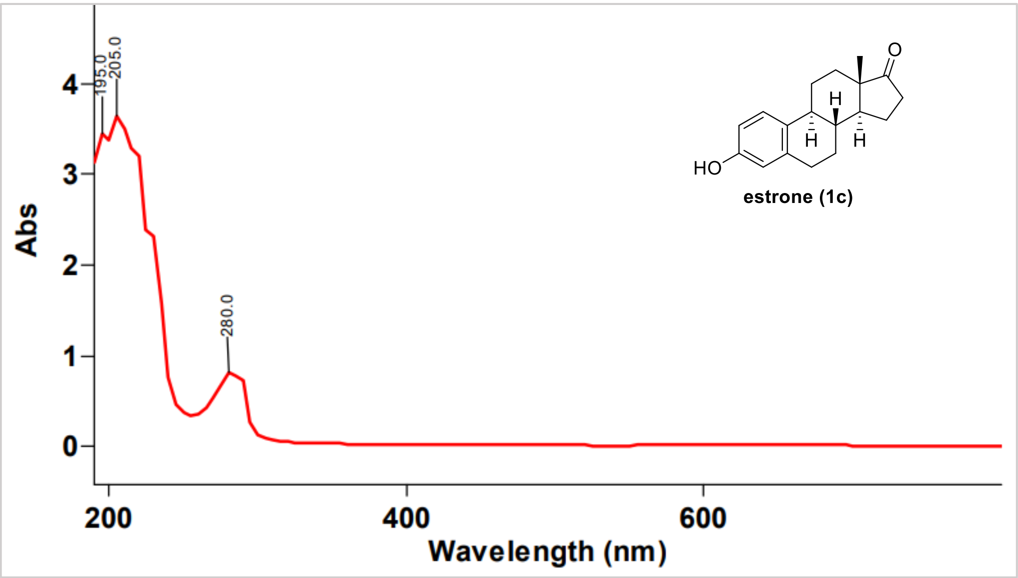

Figure S5: UV-visible spectra of estrone **1c** in 2-MeTHF.

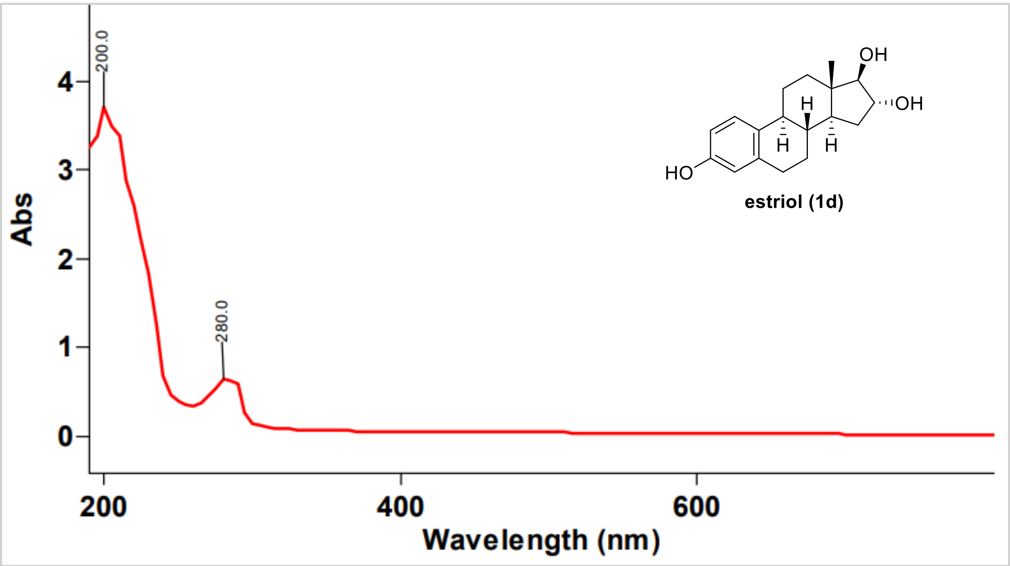

Figure S6: UV-visible spectra of estrone **1d** in 2-MeTHF.

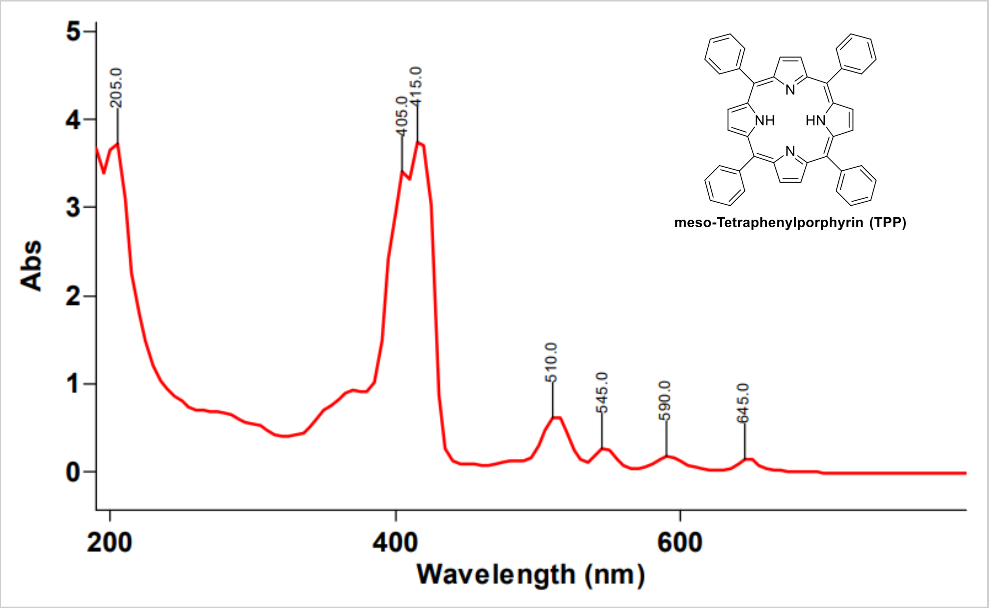

Figure S7: UV-visible spectra of meso-TPP in 2-MeTHF.

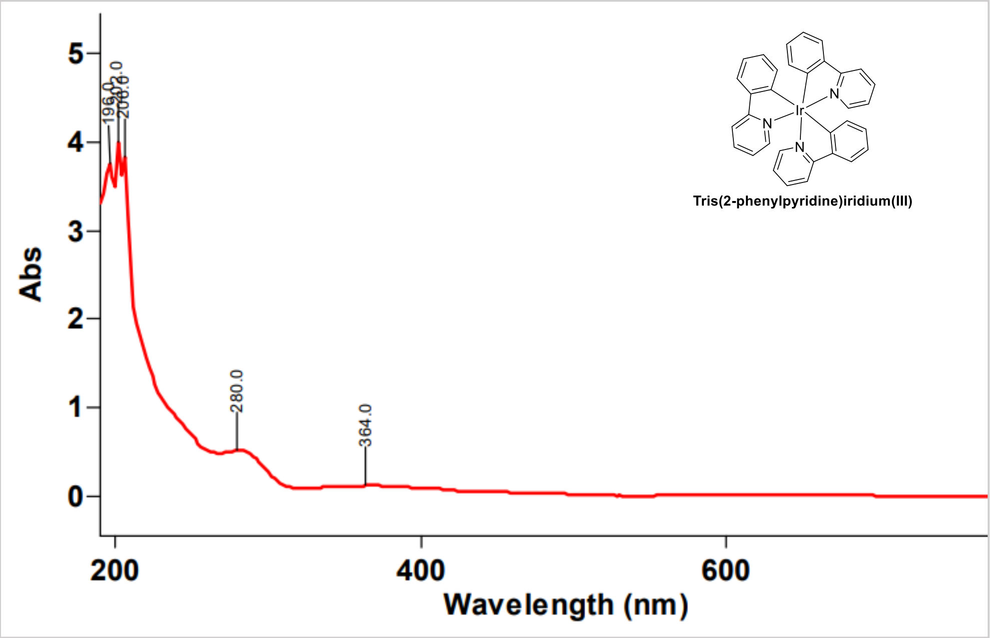

Figure S8: UV-visible spectra of $Ir(ppy)_3$ in 2-MeTHF.

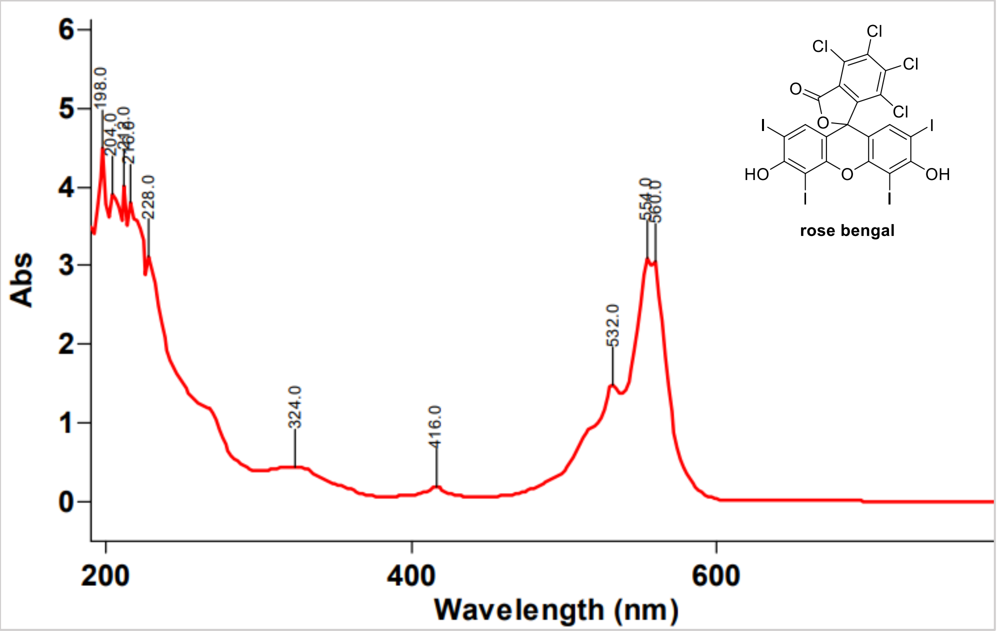

Figure S9: UV-visible spectra of rose bengal in 2-MeTHF.

4. Coumarin assay.

Coumarin (0.1 mmol) and meso-TPP (1.0 mol%) were dissolved in 2-Me-THF (1.6 mL) followed by the addition of PBS (80 µL; 0.1 M, pH 6.0) and the mixture was stirred under blue-LED irradiation and air atmosphere at 28±1 °C. At indicated intervals (1, 24 and 96 h), 100 µL of organic layer were collected, evaporated under reduced pressure and dissolved in MeOH followed by filtration of meso-TPP. The resulting sample was analysed spectrophotometrically in MeOH (6 µg/mL), using coumarin and 7-hydroxycoumarin as external standards.

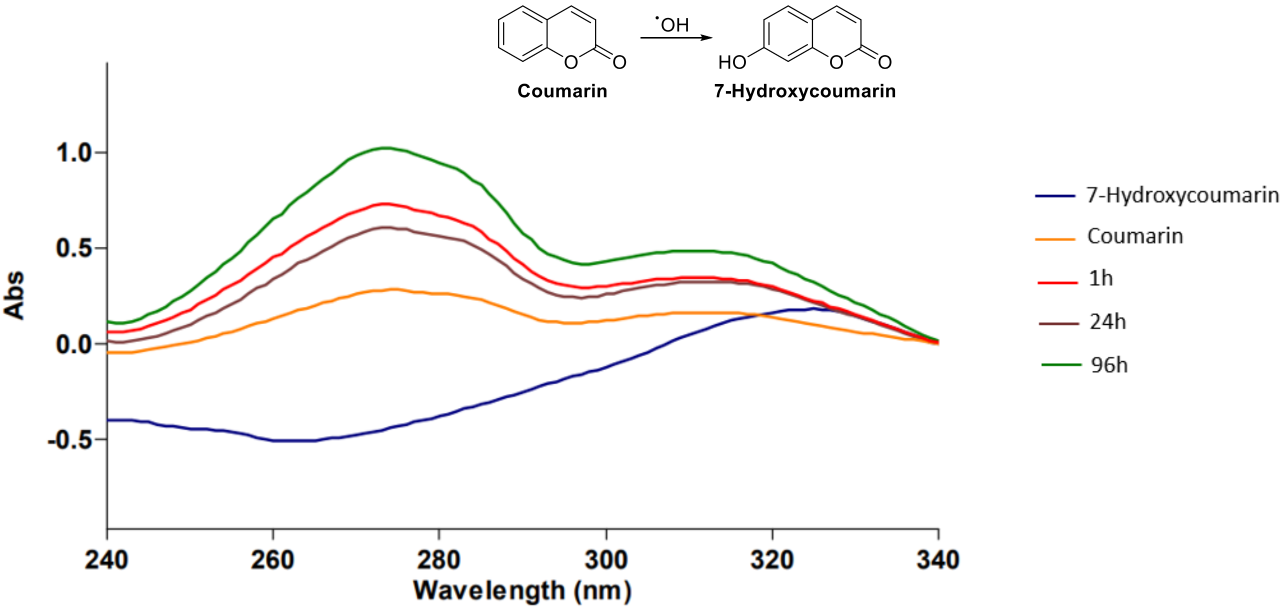

Figure S10: UV-vis analysis of the sample at different times.

5. Quenching experiments.

5.1 TEMPO assay.

Estrone **1c** (0.1 mmol) and meso-TPP (1 mol%) were dissolved in 2-Me-THF (1.6 mL) followed by the addition of PBS (0.08 mL; 0.1 M, pH 6) and TEMPO (0.2 mmol) and the mixture was gently stirred (200 rpm) under blue-LED irradiation and air atmosphere at 28 ± 1 °C for 24 hrs. The reaction mixture was then washed with brine (3×2 mL), dried over sodium sulphate and evaporated under vacuum. The hydroperoxide **2c** was isolated in 71% of yield after purification by column chromatography (R_f = 0.21; PE:AcOEt 3:2).

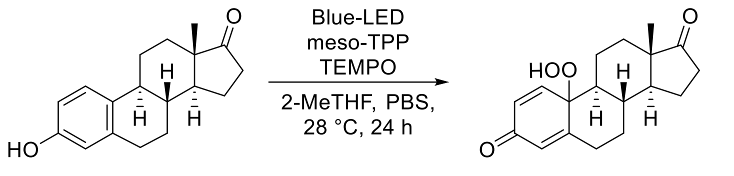

Figure S11: Photo-oxygenation of estrone **1**c in presence of TEMPO as radical scavenger.

5.2 NaN_3 assay.

Estrone **1c** (0.1 mmol) and meso-TPP (1 mol%) were dissolved in 2-Me-THF (1.6 mL) followed by the addition of PBS (0.08 mL; 0.1 M, pH 6) and NaN_3 (1.0 mmol) and the mixture was gently stirred (200 rpm) under blue-LED irradiation and air atmosphere at 28 ± 1 °C for 24 hrs. The reaction mixture was washed with brine (3×2 mL), dried over sodium sulphate and evaporated under vacuum. The hydro-peroxide **2c** was isolated in 30% of yield after purification by column chromatography (R_f = 0.21; PE:AcOEt 3:2).

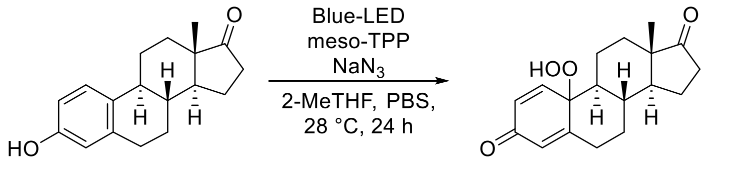

Figure S12: Photo-oxygenation of estrone **1**c in presence of NaN_3 as singlet oxygen scavenger.

6. Photooxygenation of estrogens under argon atmosphere and degassed solvents.

A) Degassing of 2-MeTHF (freeze-pump-thaw method):

2-MeTHF was added in a Schlenk flask under argon atmosphere and freezed in liquid nitrogen. The stopcock was open and keep under vacuum for 3-5 minutes whilst submerged in liquid nitrogen to evacuate the headspace. The latter procedure was repeated overall for three times. At the end the shlenk was removed from the dewar and the solvent heated at room temperature. The overall process was repeated two more times.

B) Degassing of PBS (Argon/Ultrasonic degassing method):

The PBS was degassed by placing the flask containing the aqueous phase in an ultrasonic bath and bubbling argon into the liquid for 1.0 hrs. Oxygen trapped in the liquid was vented with a vent valve.

Reaction set-up: 17α-ethinylestradiol **1b** (0.1 mmol) and meso-TPP (1 mol%) were dissolved in degassed 2-Me-THF (1.6 mL) followed by addition of degassed PBS (80 µL, 0.1 M, pH 6) and the mixture was gently stirred (200 rpm) under blue-LED irradiation and argon atmosphere at 28 ± 1 °C for 24 hrs. At indicated times (1, 24, 48 h), the reaction was monitored through thin layer chromatography (TLC). No conversion of substrate was detected.

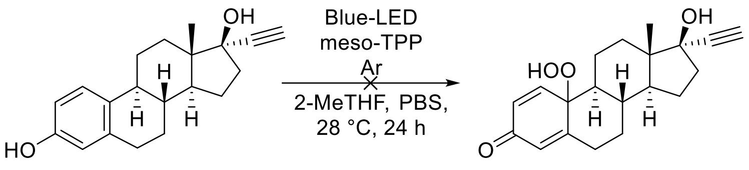

1b **2b**

Figure S13: Photo-oxygenation of 17α-ethinylestradiol **1b** under an argon atmosphere and with degassed solvents.

7. Screening of reducing agents.

7.1. $Na_2S_2O_3$.

Hydro-peroxide **2b** (0.1 mmol) was solubilized in MeOH and water (4:1 v/v, 6 mL) followed by the addition of $Na_2S_2O_3$ (1.0 mmol). The reaction was stirred at room temperature overnight and evaporated under reduced pressure. The crude was solubilized in AcOEt (6 mL) and washed with water (2 x 3 mL) and brine (3 mL) and the organic layer was dried over Na_2SO_4. After evaporation under reduced pressure the crude was analysed by ^{13}C NMR showing a partial conversion of substrate associated to the contemporary presence of the representative C-10 signal of the substrate (81.020 ppm) and of the product (69.632 ppm).

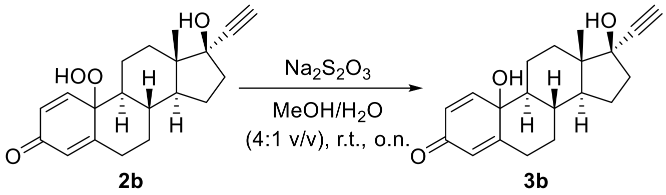

Figure S14: Reduction of hydro-peroxide **2b** by $Na_2S_2O_3$.

7.2. KI.

Hydro-peroxide **2b** (0.1 mmol) was solubilized in EtOH (1 mL) followed by the addition of aqueous solution of KI (1M, 2 mL). The reaction was stirred at room temperature overnight and evaporated under reduced pressure. The crude was solubilized in AcOEt (6 mL) and washed with water (2 x 3 mL) and brine (3 mL), and the organic layer was dried over Na_2SO_4. After evaporation under reduced pressure the crude product was analysed by ^{13}C NMR. No substrate conversion was detected.

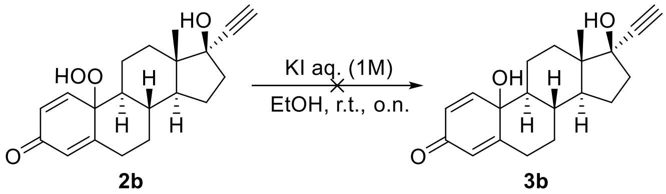

Figure S15: Reduction of hydro-peroxide **2b** by KI.

7.3. PPh_3.

Hydro-peroxide **2b** (0.1 mmol) was solubilized in EtOH (0.5 mL) followed by the addition of PPh_3 (0.15 mmol). The reaction was stirred at room temperature overnight and evaporated under reduced pressure. The crude was solubilized in AcOEt (6 mL) and washed with water (2 x 3 mL) and brine (3 mL), and the organic layer was dried over Na_2SO_4. After evaporation under reduced pressure the crude product was analysed by ^{13}C NMR. The substrate was quantitatively converted and quinol **3b** was obtained in quantitative yield.

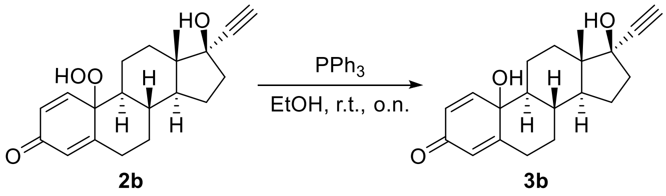

Figure S16: Reduction of hydro-peroxide **2b** by PPh_3.

8. NMR spectroscopy of hydroperoxides 2a-d and quinols 3a-d

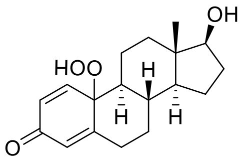

2a

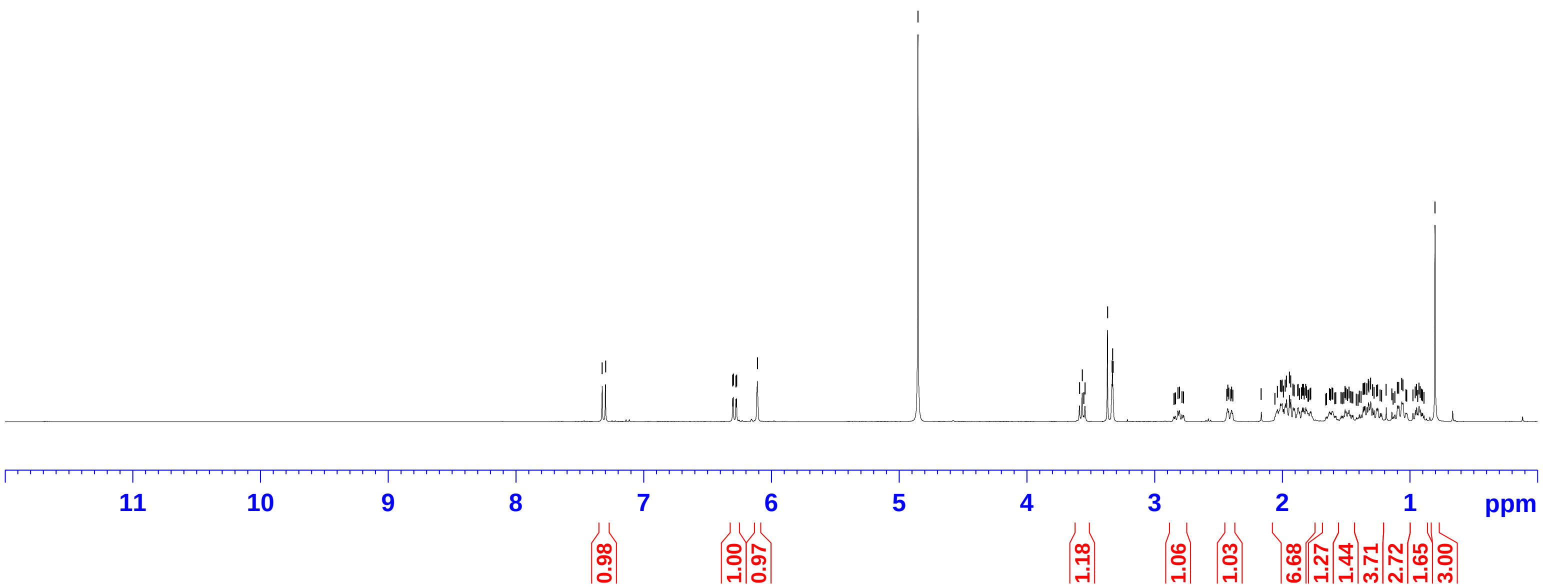

Figure S17: ^{1}H NMR (CD_3OD, 400 MHz) of compound **2a**.

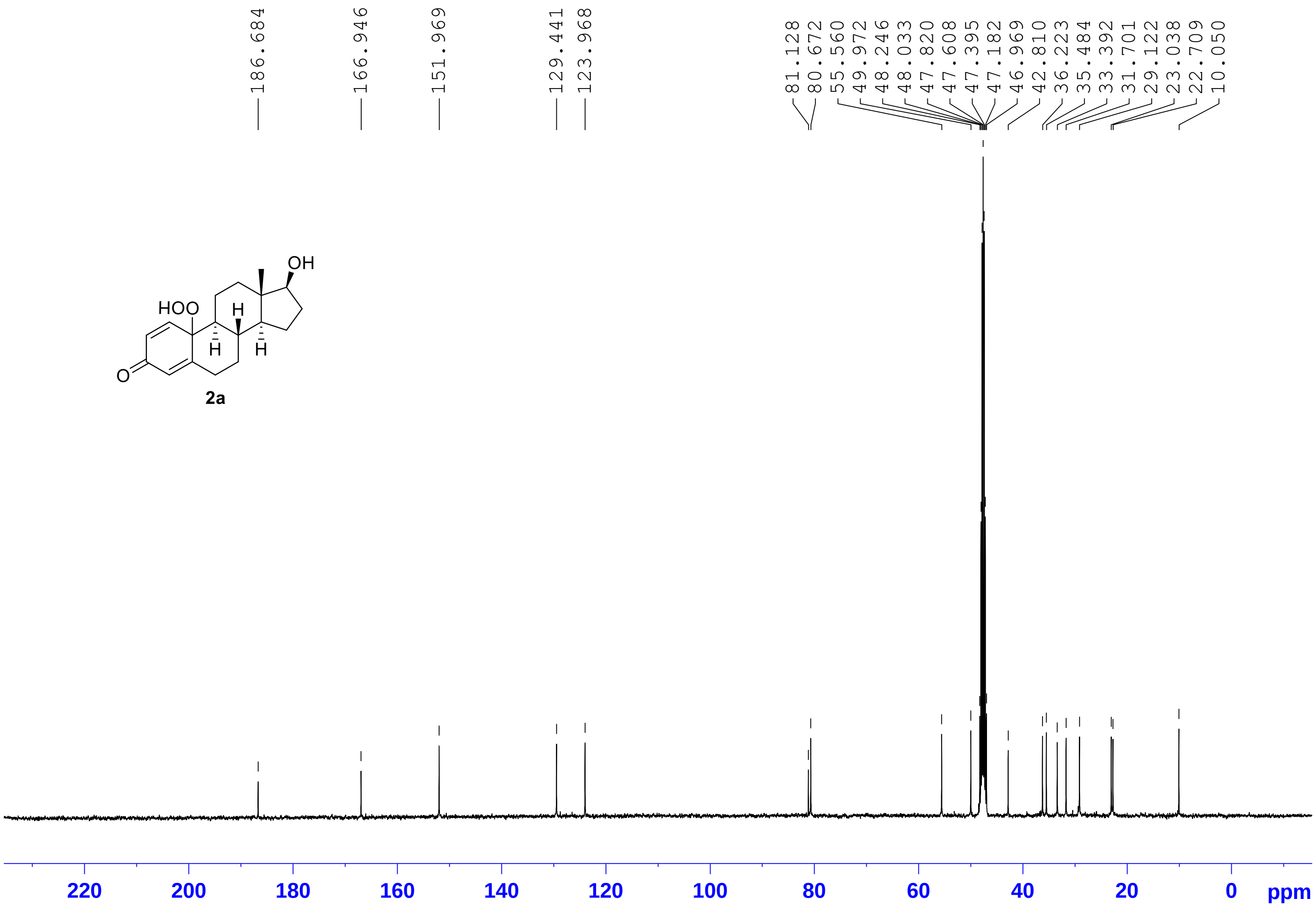

Figure S18: ^{13}C NMR (CD_3OD, 100 MHz) of compound **2a**.

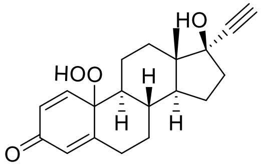

2b

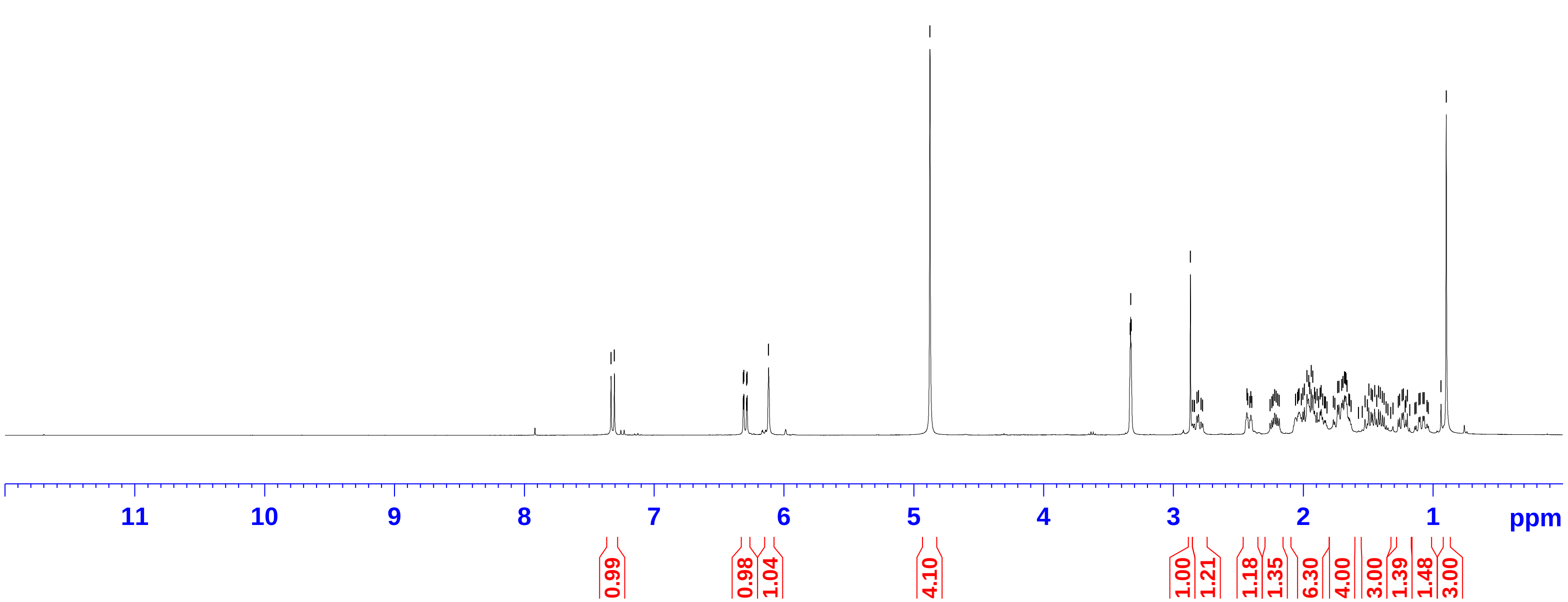

Figure S19: ^{1}H NMR (CD_3OD, 400 MHz) of compound **2b**.

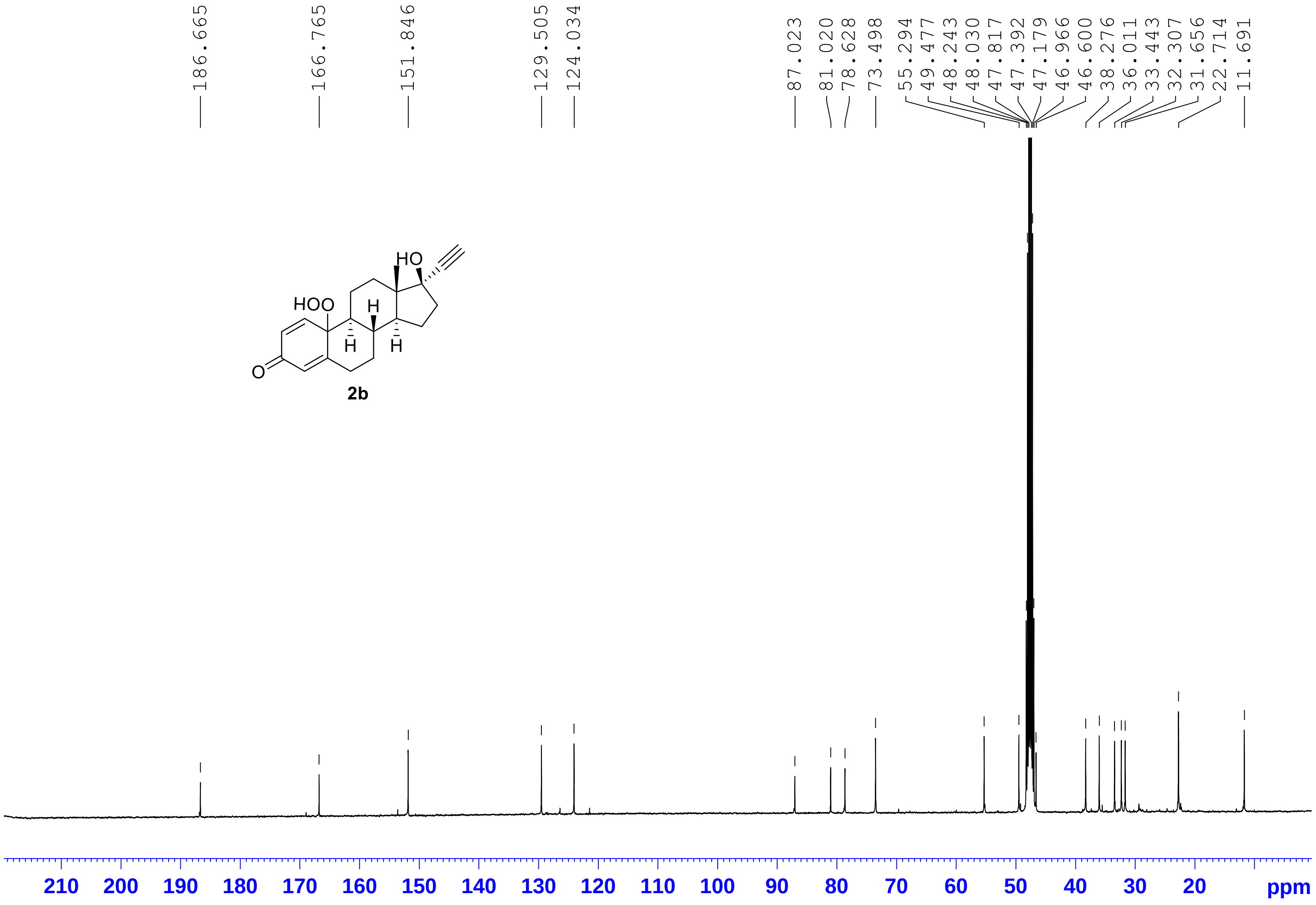

Figure S20: ^{13}C NMR (CD_3OD, 100 MHz) of compound **2b**.

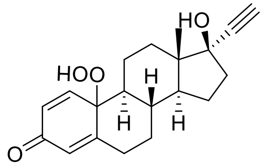

2b

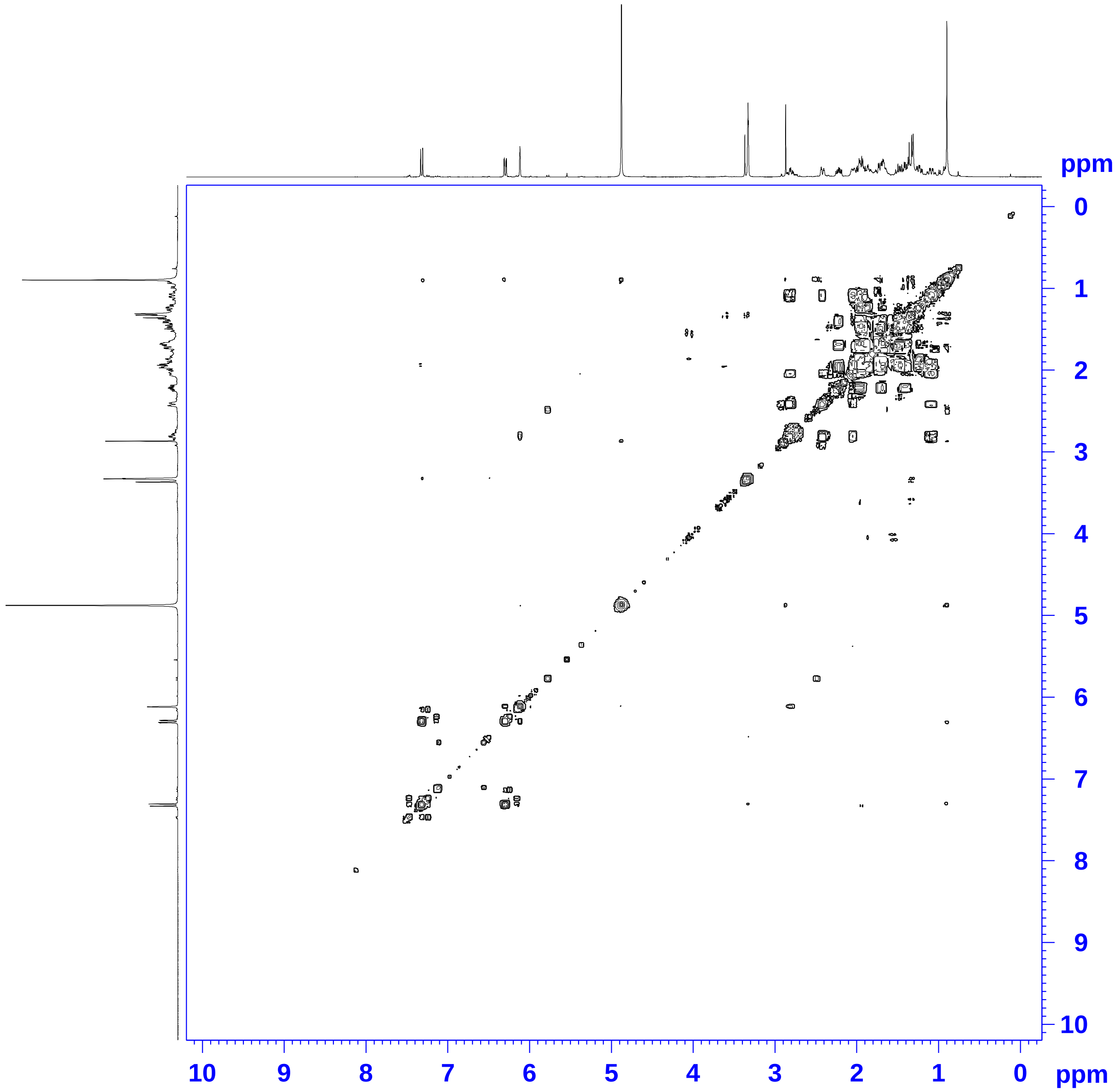

Figure S21: COSY (CD_3OD, 400 MHz) of compound **2b**.

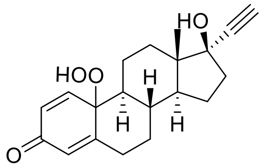

2b

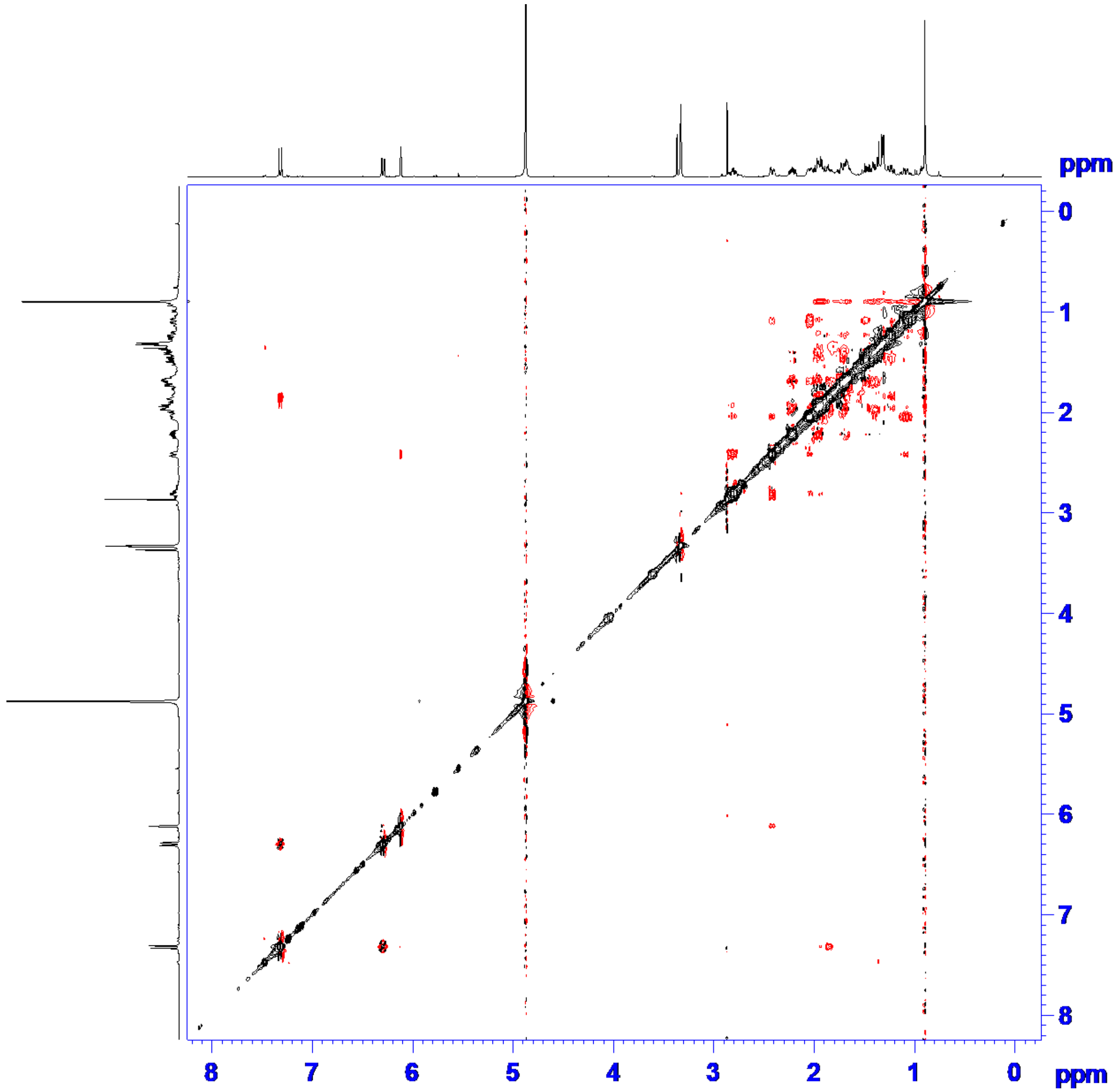

Figure S22: NOESY (CD_3OD, 400 MHz) of compound **2b**.

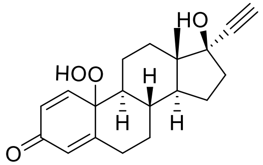

2b

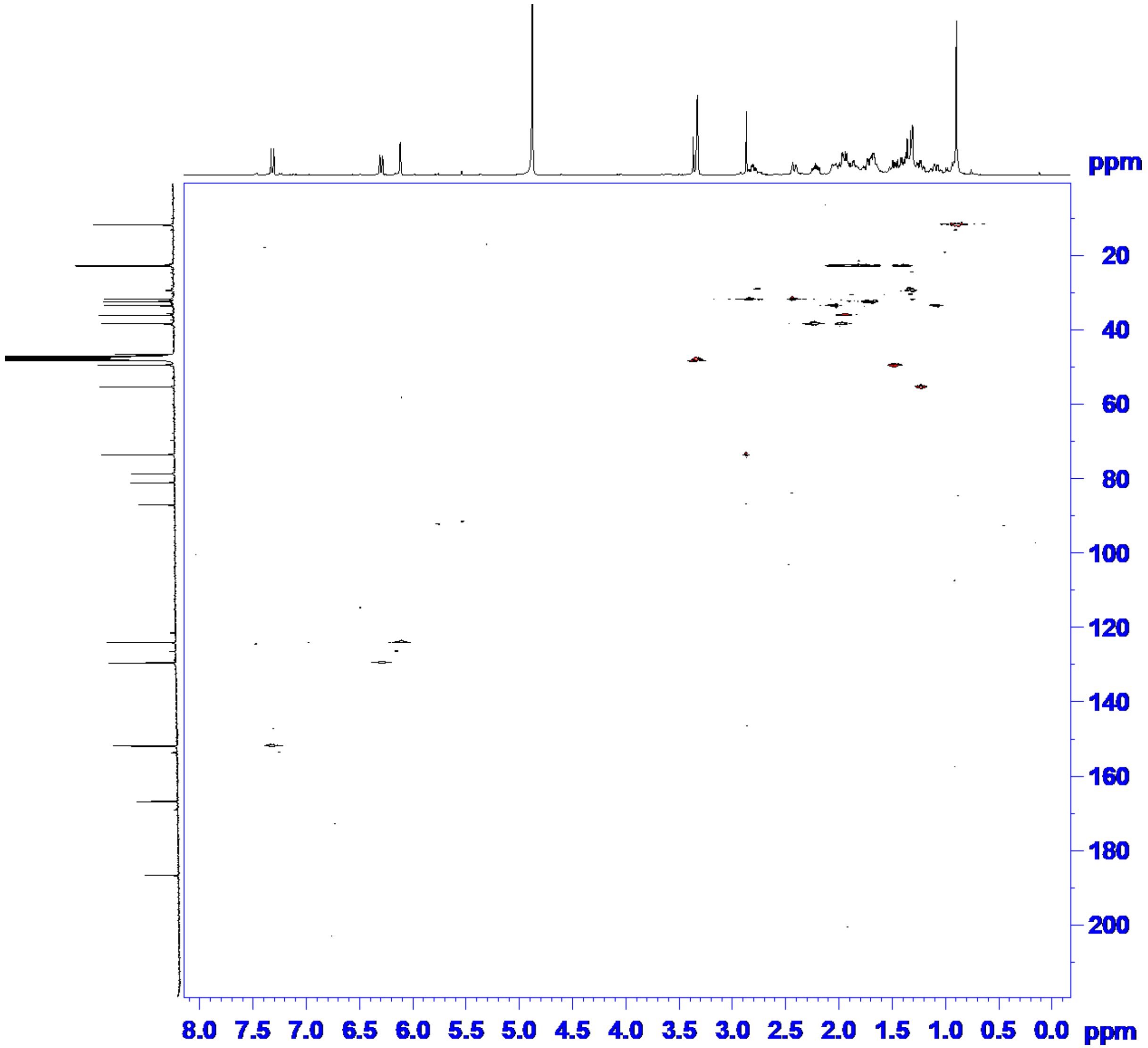

Figure S23: HSQC (CD_3OD, 400 MHz) of compound **2b**.

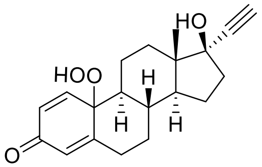

2b

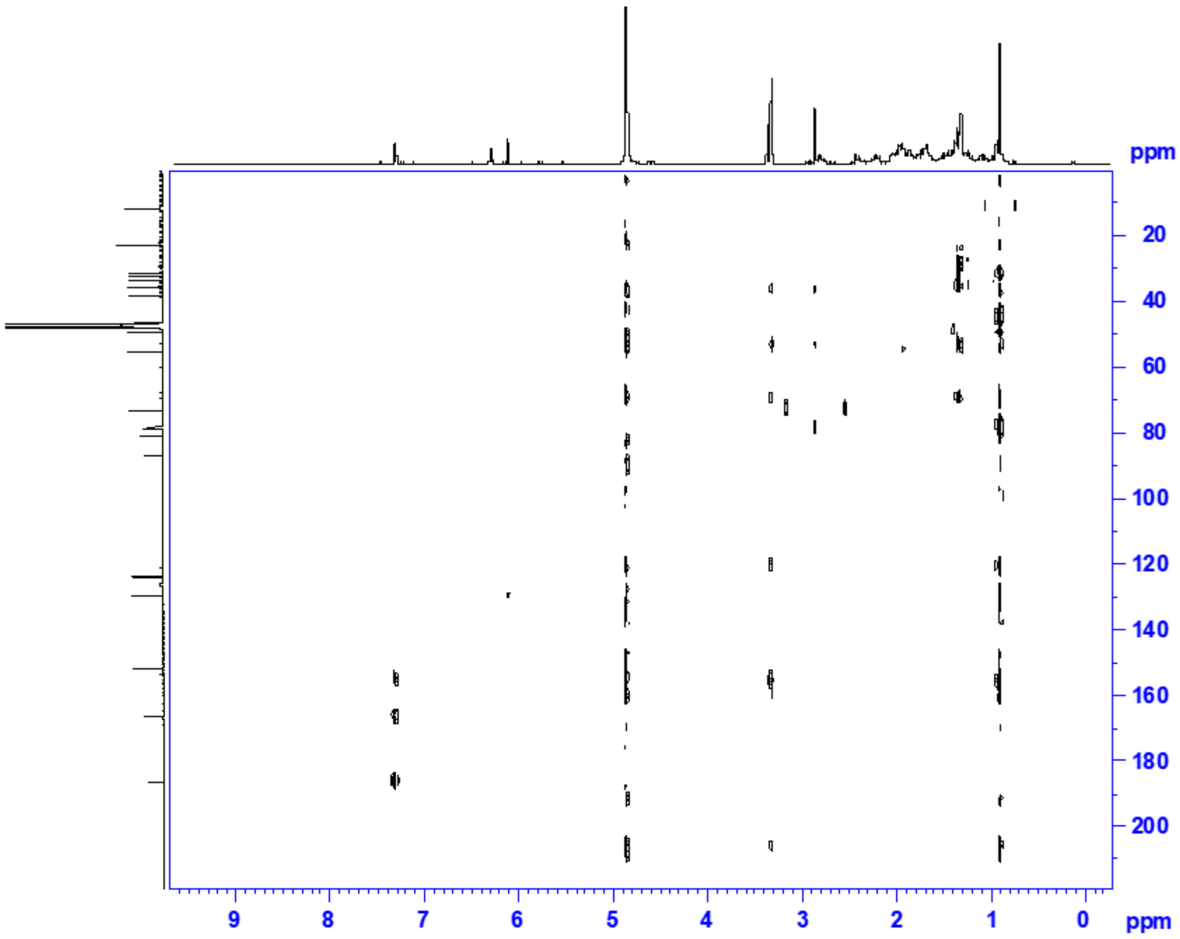

Figure S24: HMBC (CD_3OD, 400 MHz) of compound **2b**.

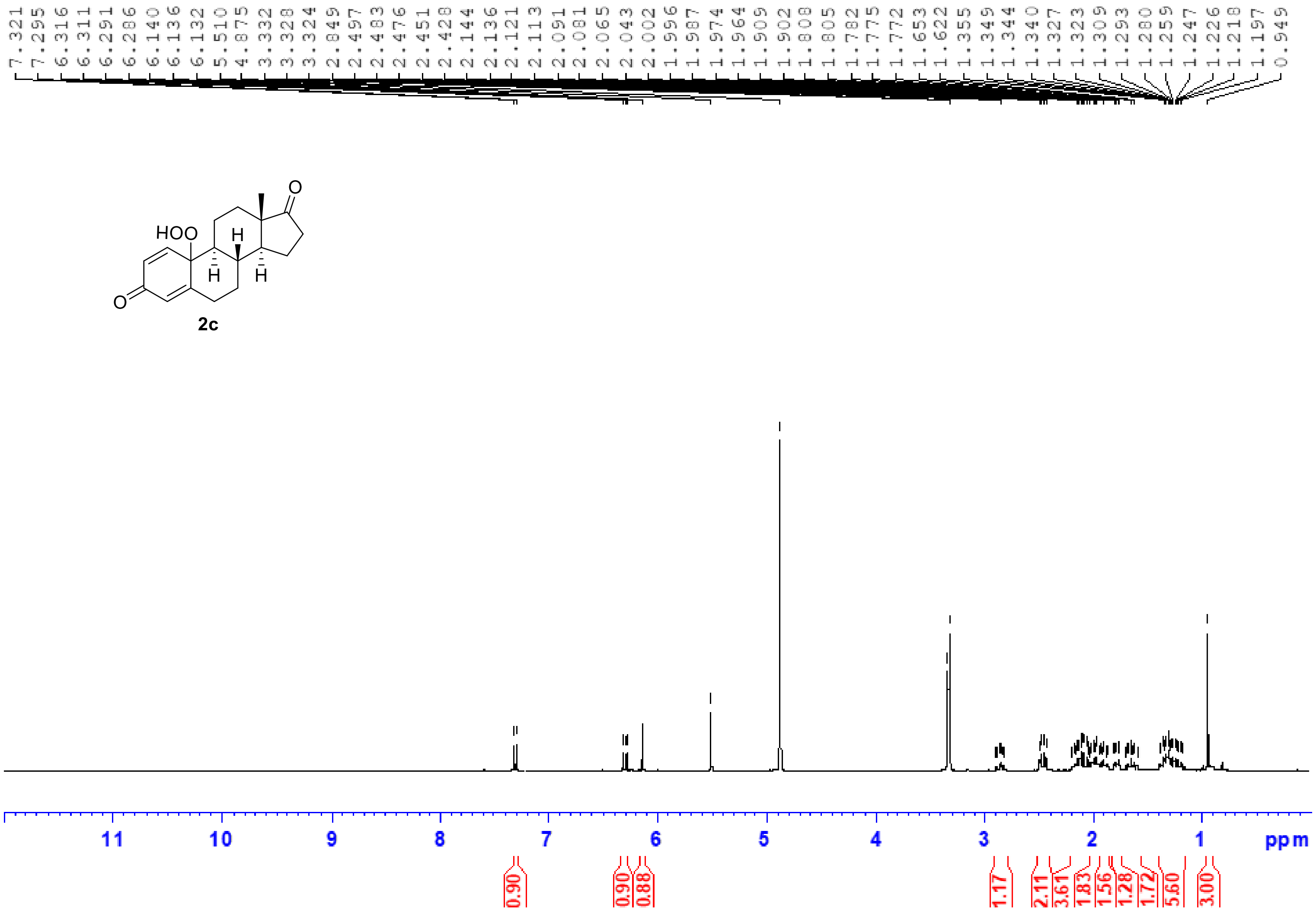

Figure S25: ^{1}H NMR (CD_3OD, 400 MHz) of compound **2c**.

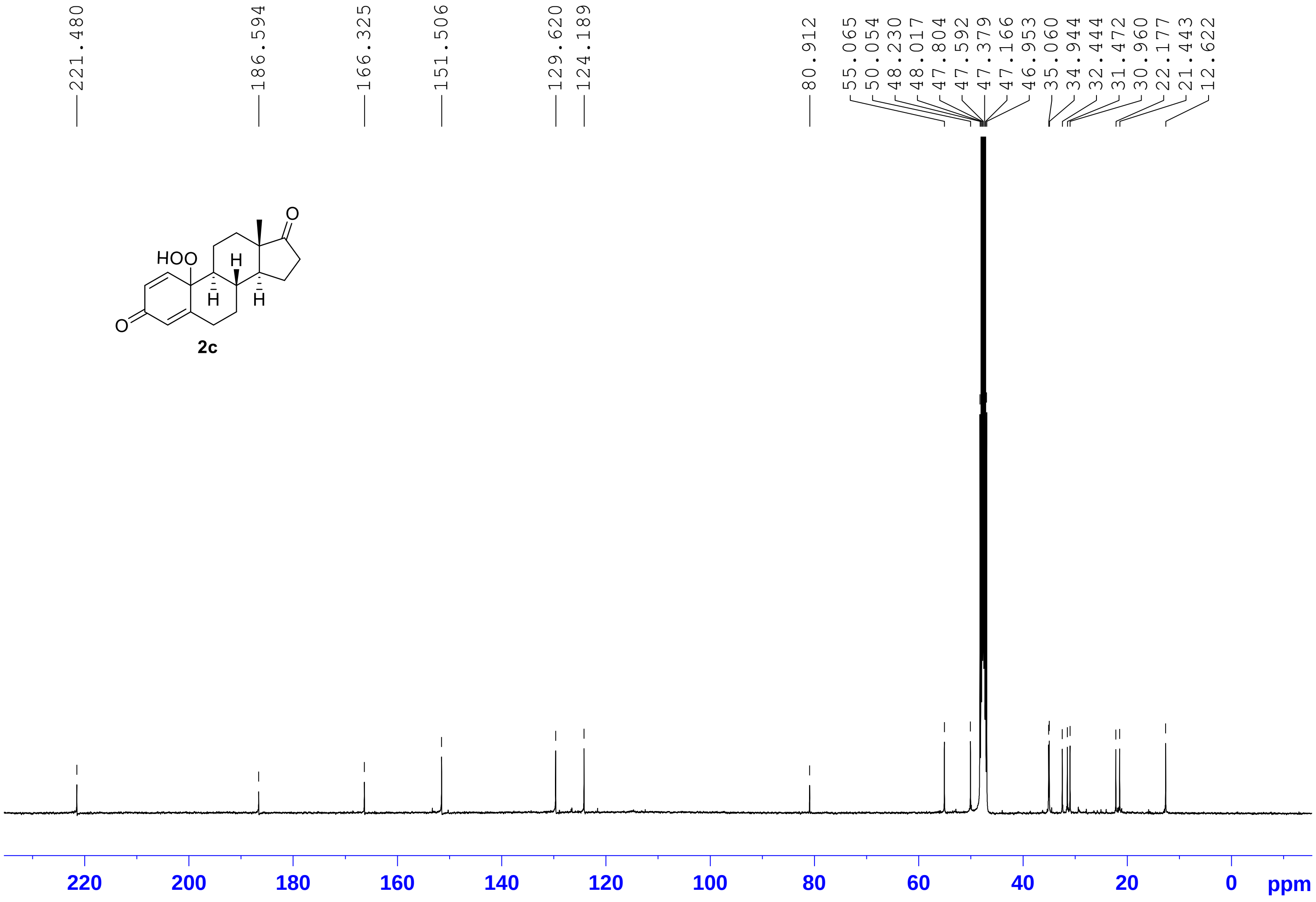

Figure S26: ^{13}C NMR (CD_3OD, 100 MHz) of compound **2c**.

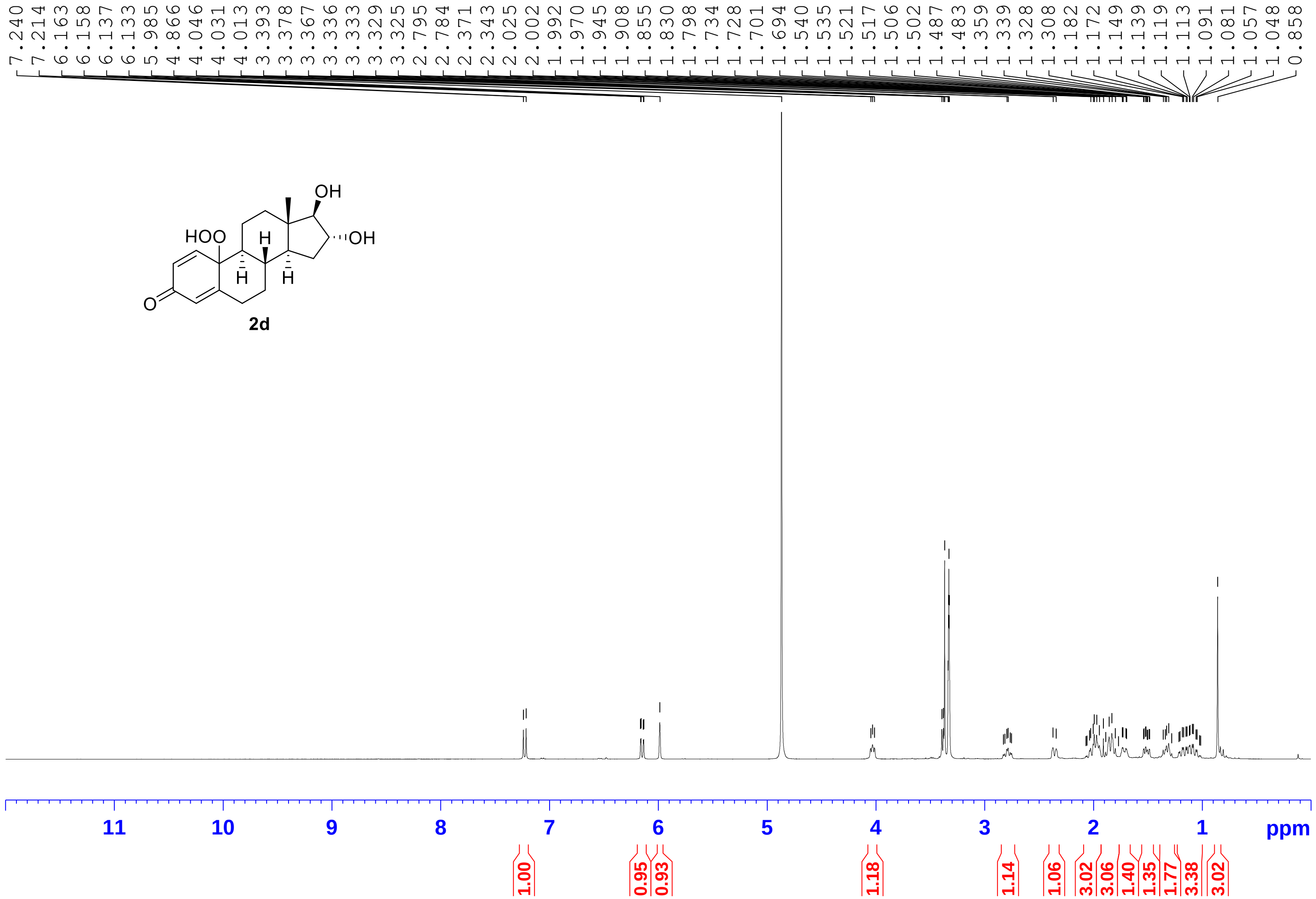

Figure S27: ^{1}H NMR (CD_3OD, 400 MHz) of compound **2d**.

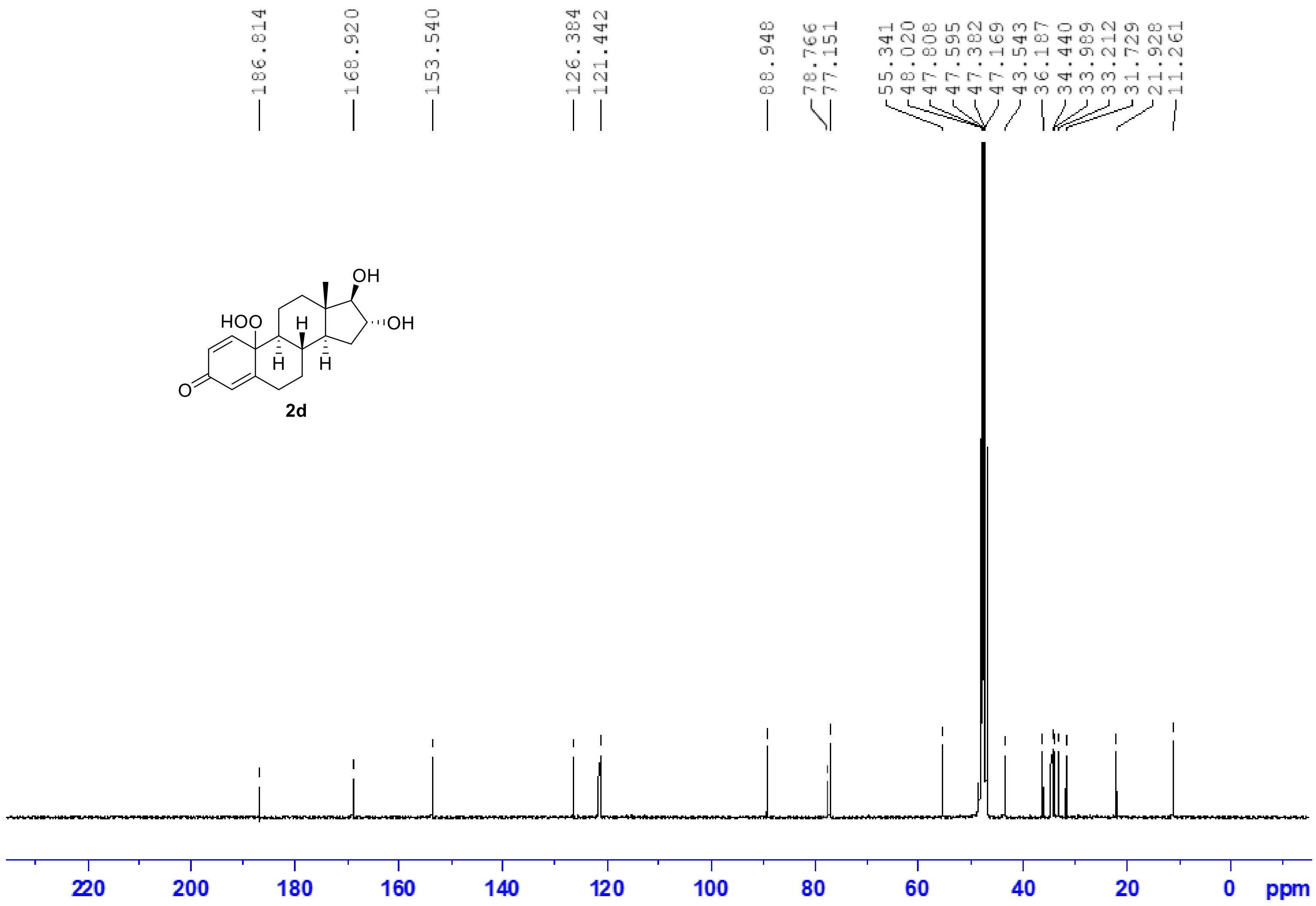

Figure S28: ^{13}C NMR (CD_3OD, 100 MHz) of compound **2d**.

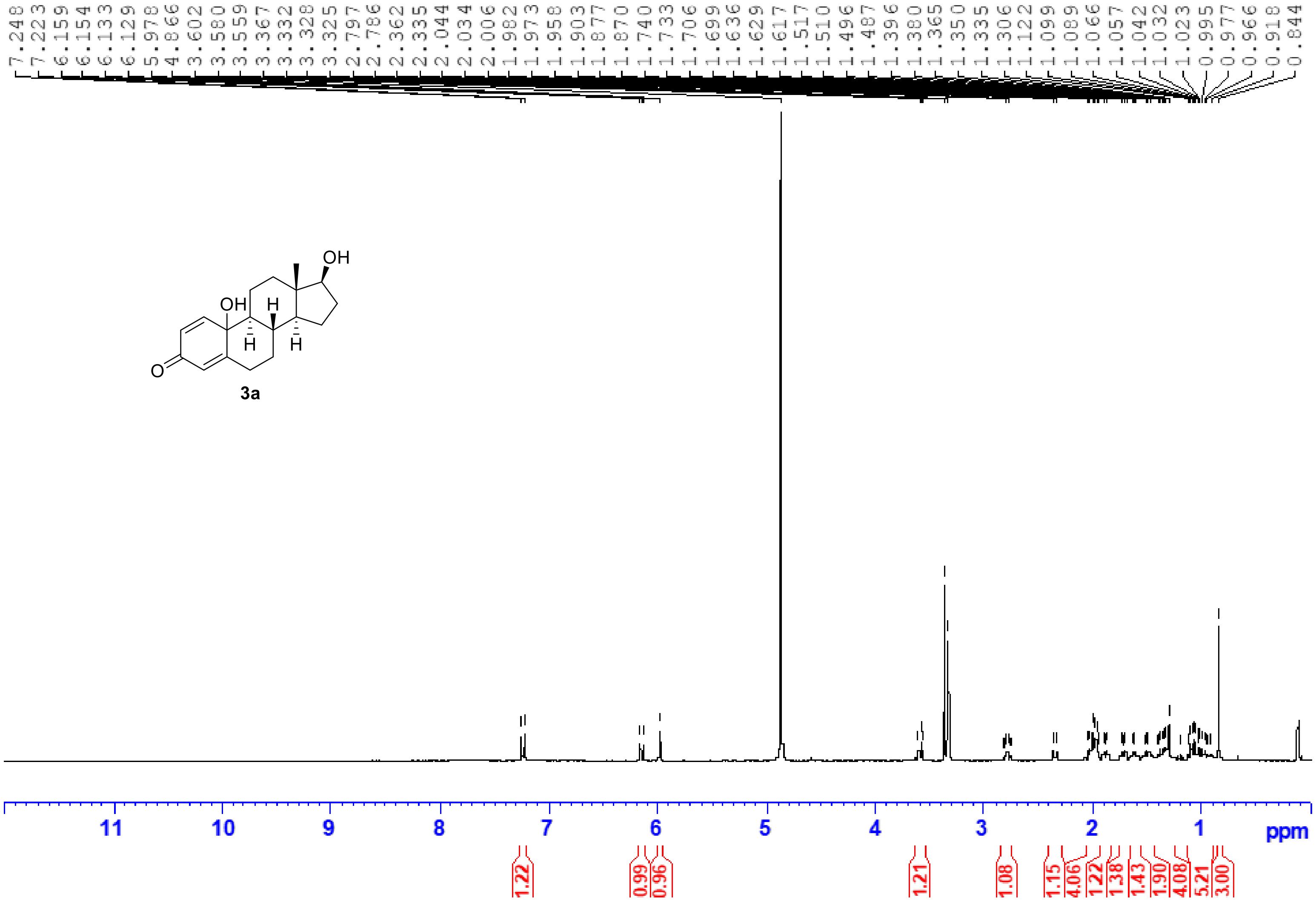

Figure S29: ^{1}H NMR (CD_3OD, 400 MHz) of compound **3a**.

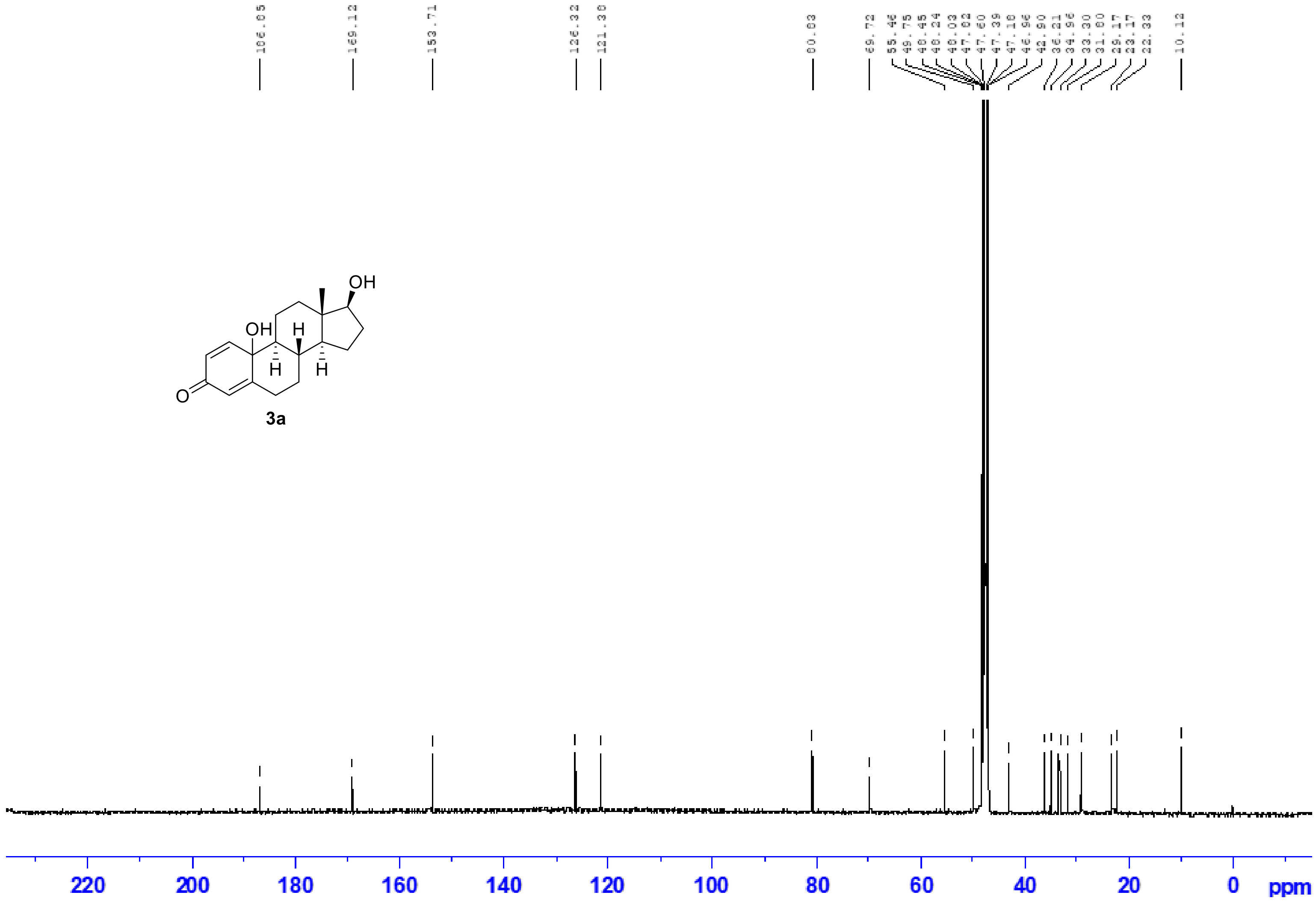

Figure S30: ^{13}C NMR (CD_3OD, 100 MHz) of compound **3a**.

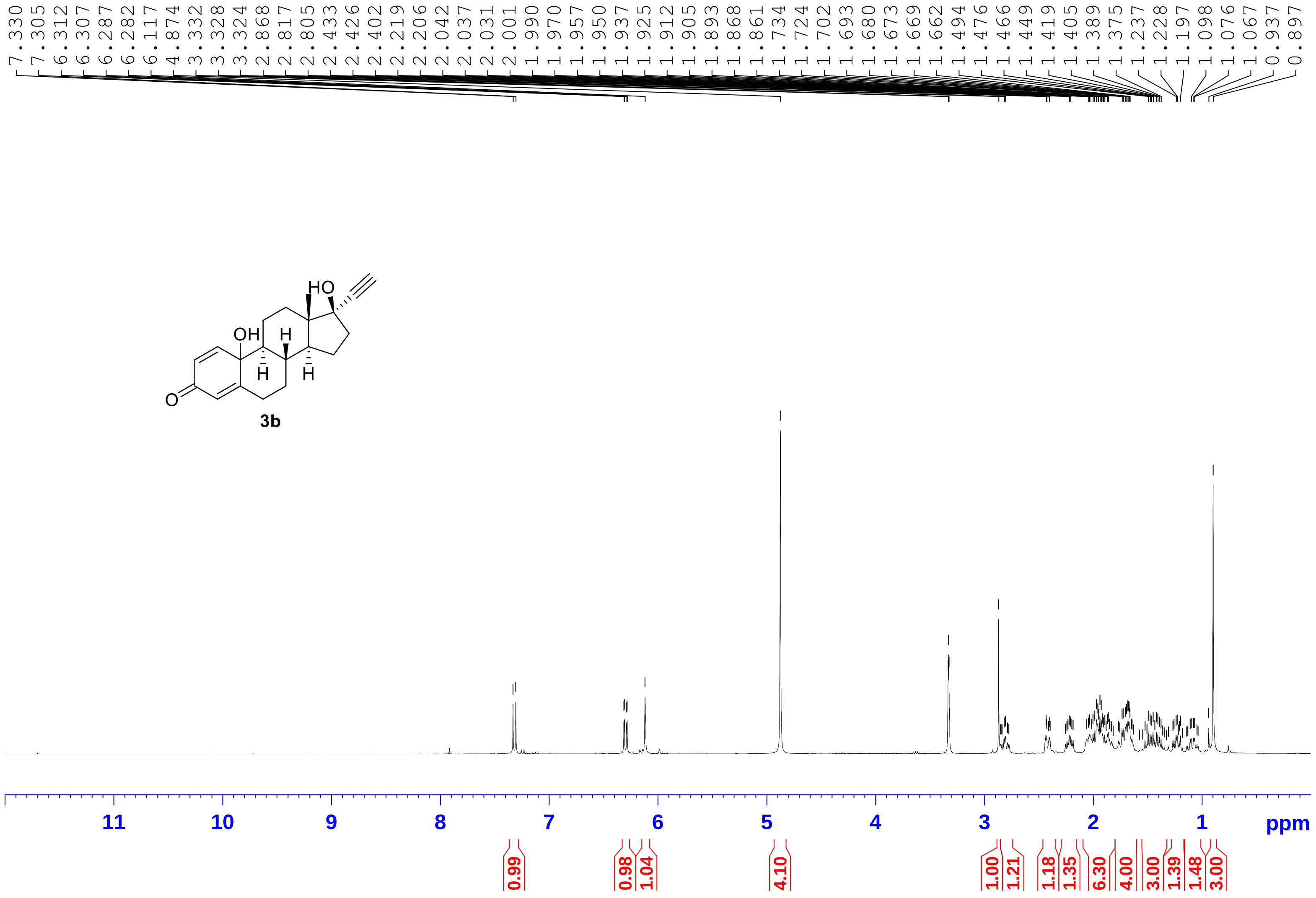

Figure S31: ^{1}H NMR (CD_3OD, 400 MHz) of compound **3b**.

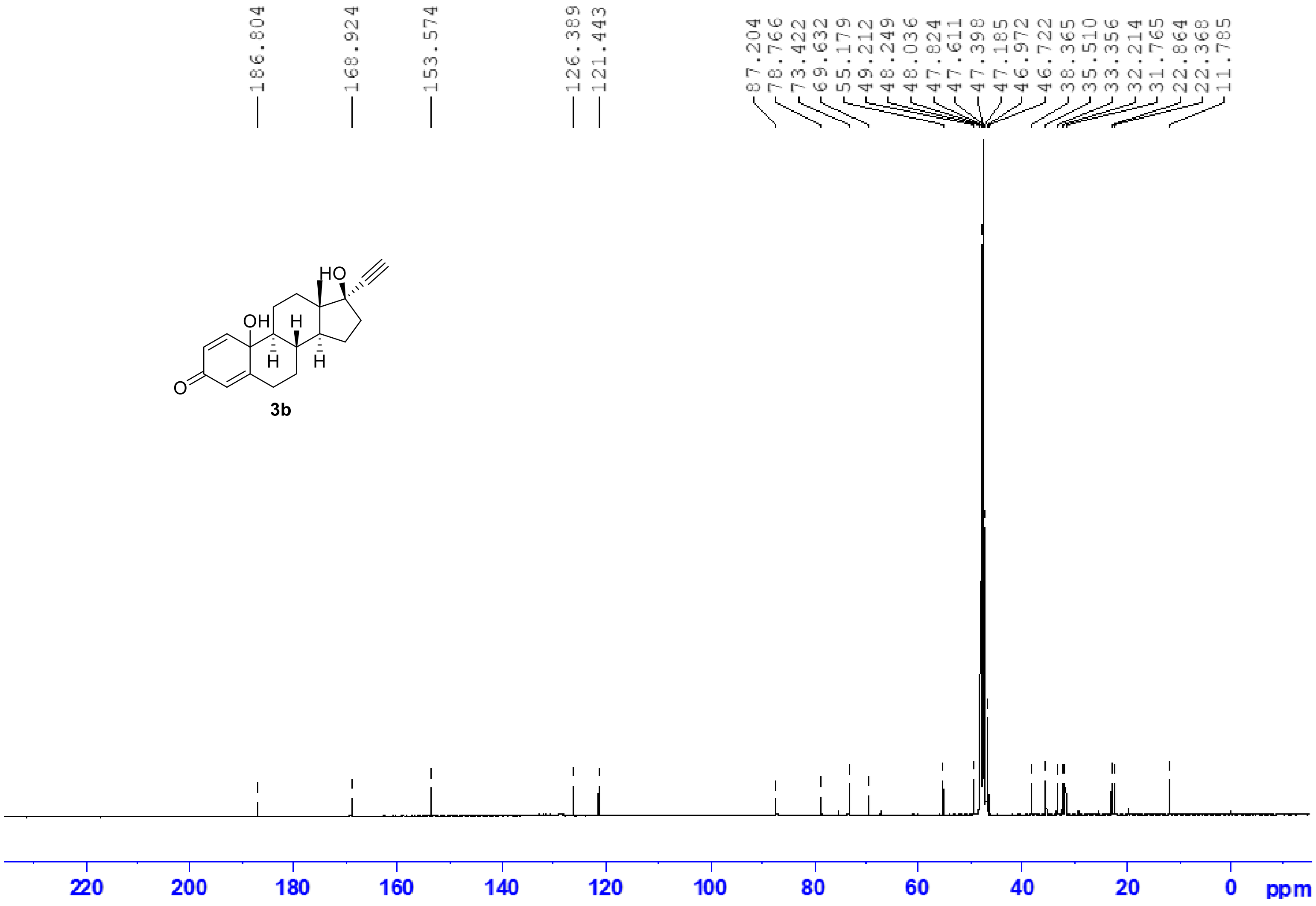

Figure S32: ^{13}C NMR (CD_3OD, 100 MHz) of compound **3b**.

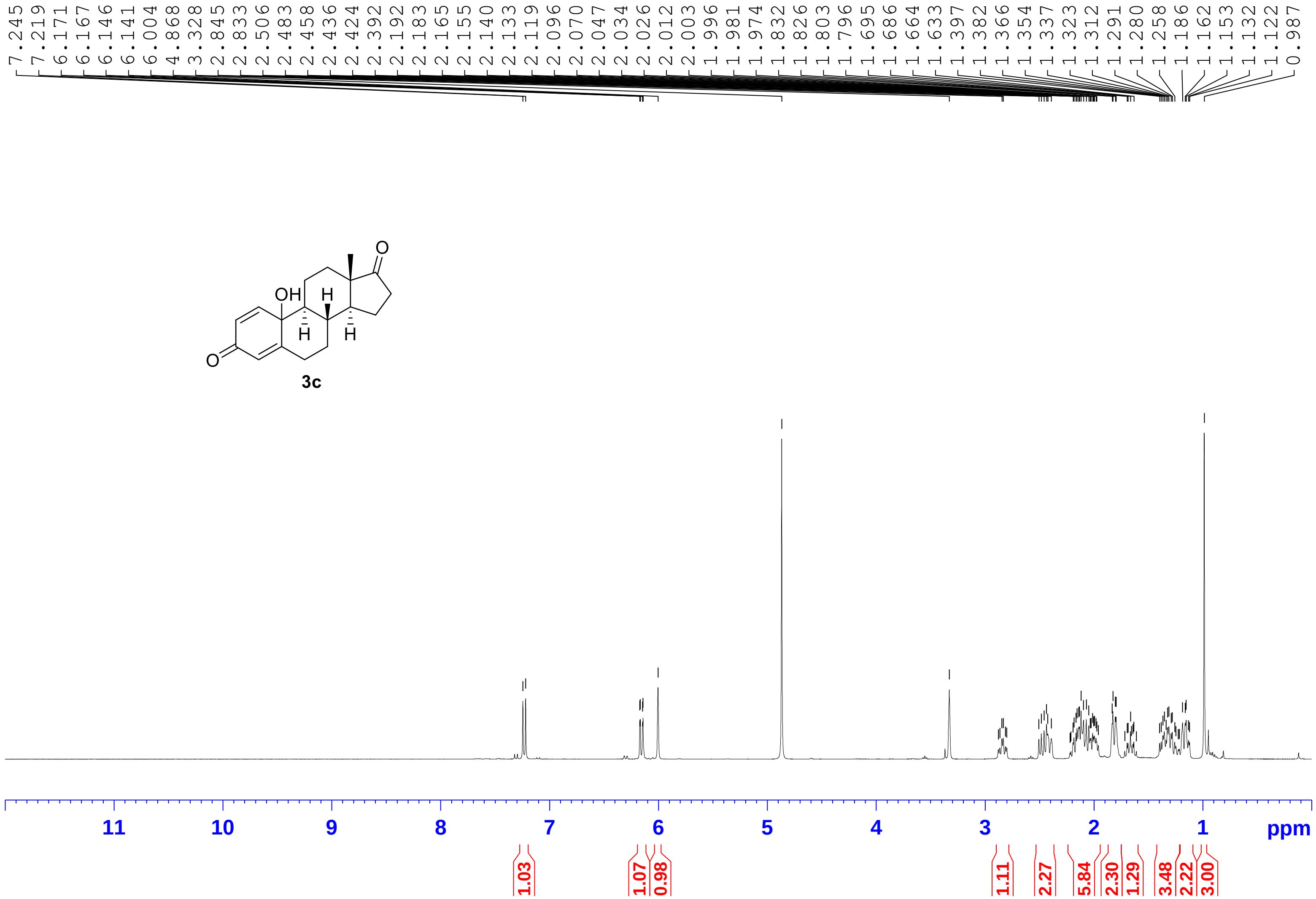

Figure S33: ^{1}H NMR (CD_3OD, 400 MHz) of compound **3c**.

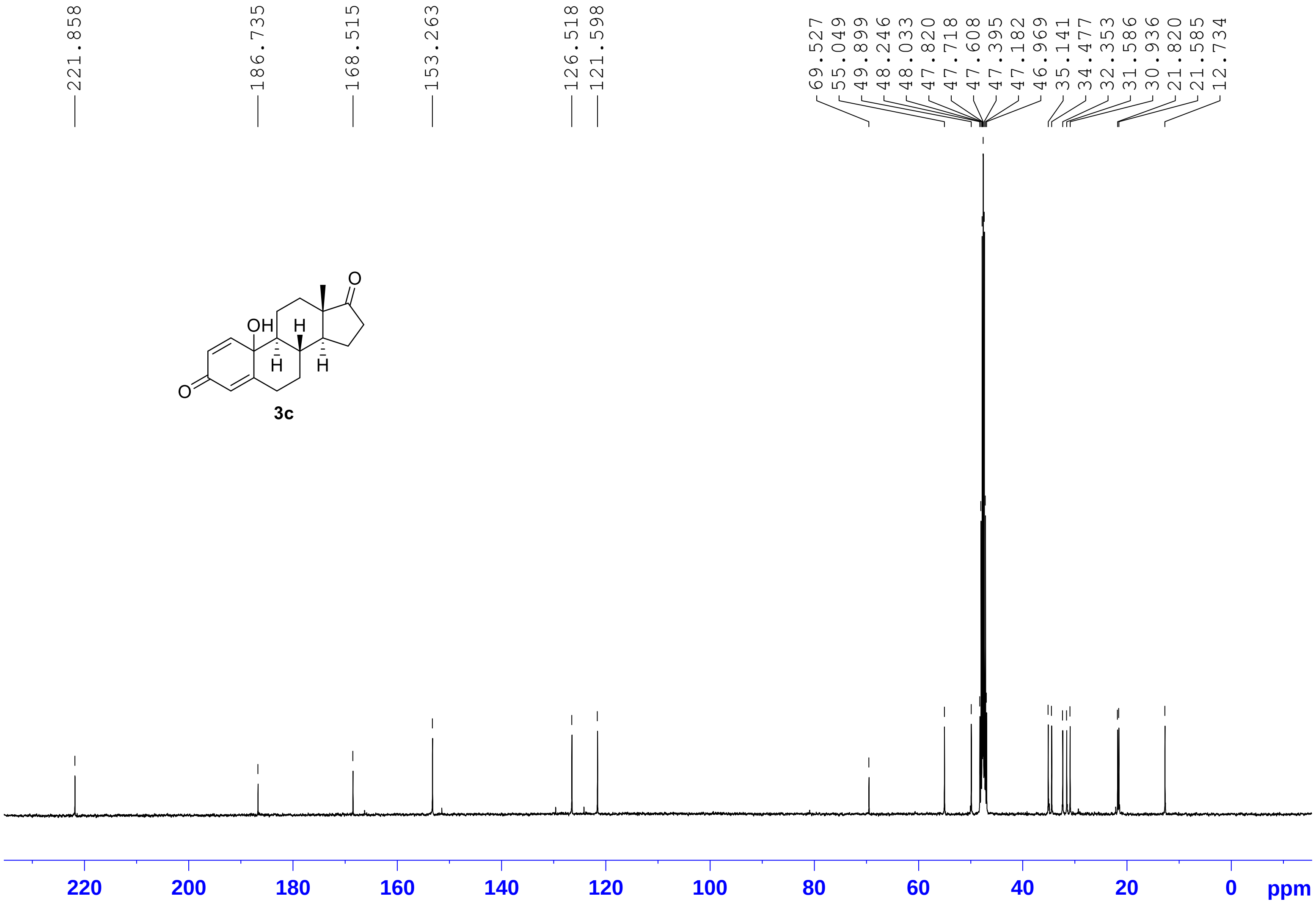

Figure S34: ^{13}C NMR (CD_3OD, 100 MHz) of compound **3c**.

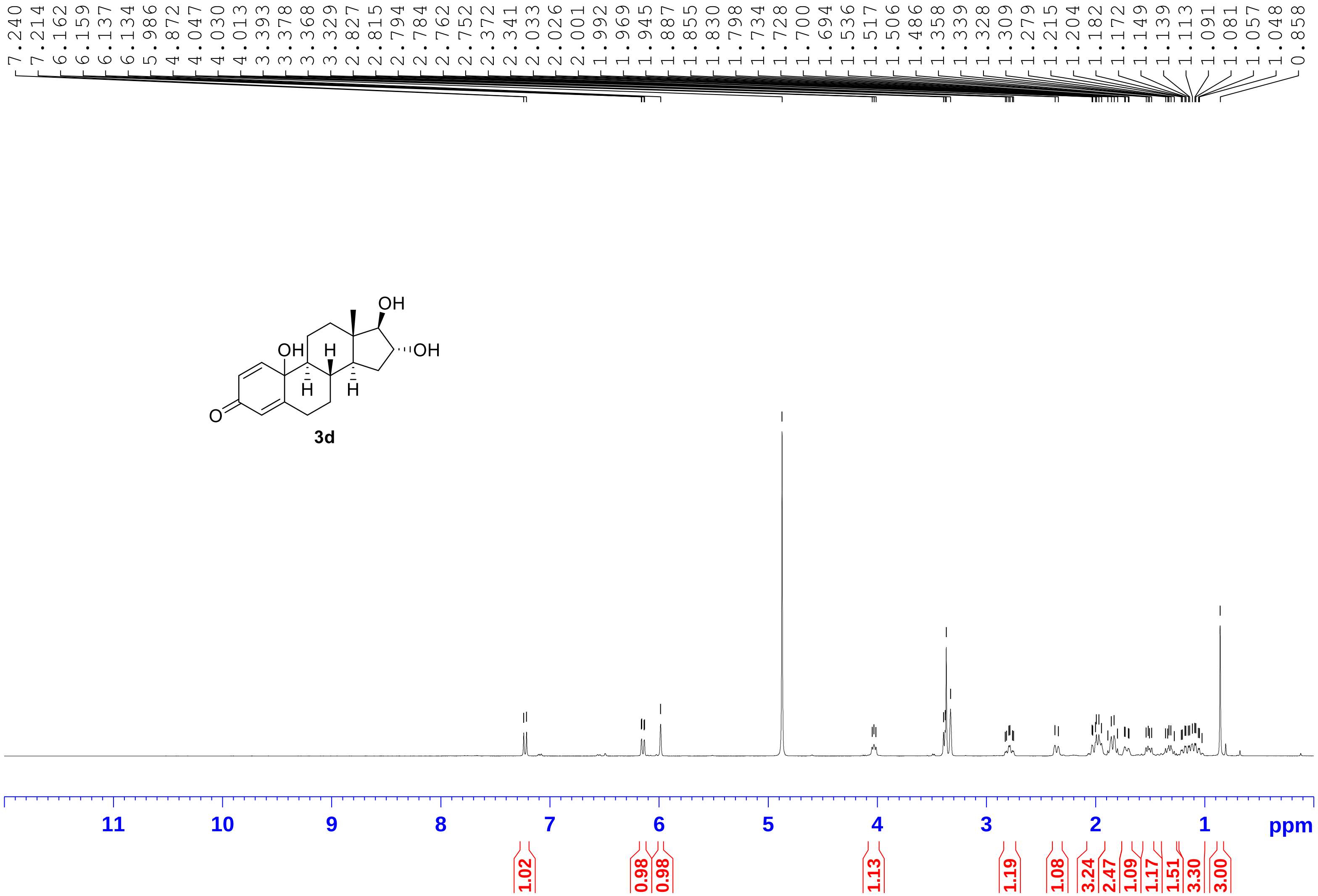

Figure S35: [13]H NMR (CD_3OD, 400 MHz) of compound **3d**.

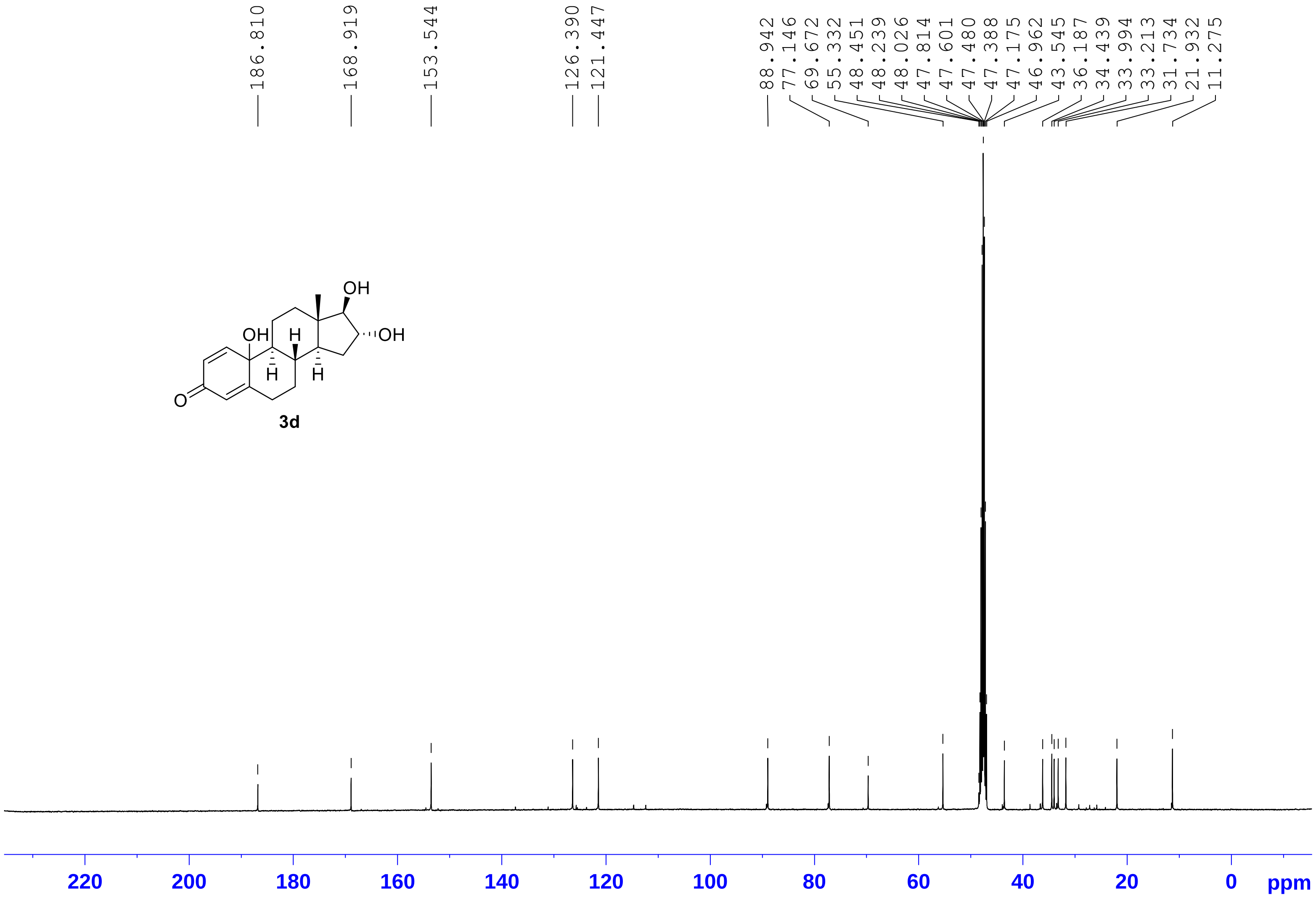

Figure S36: ^{13}C NMR (CD_3OD, 100 MHz) of compound **3d**.
